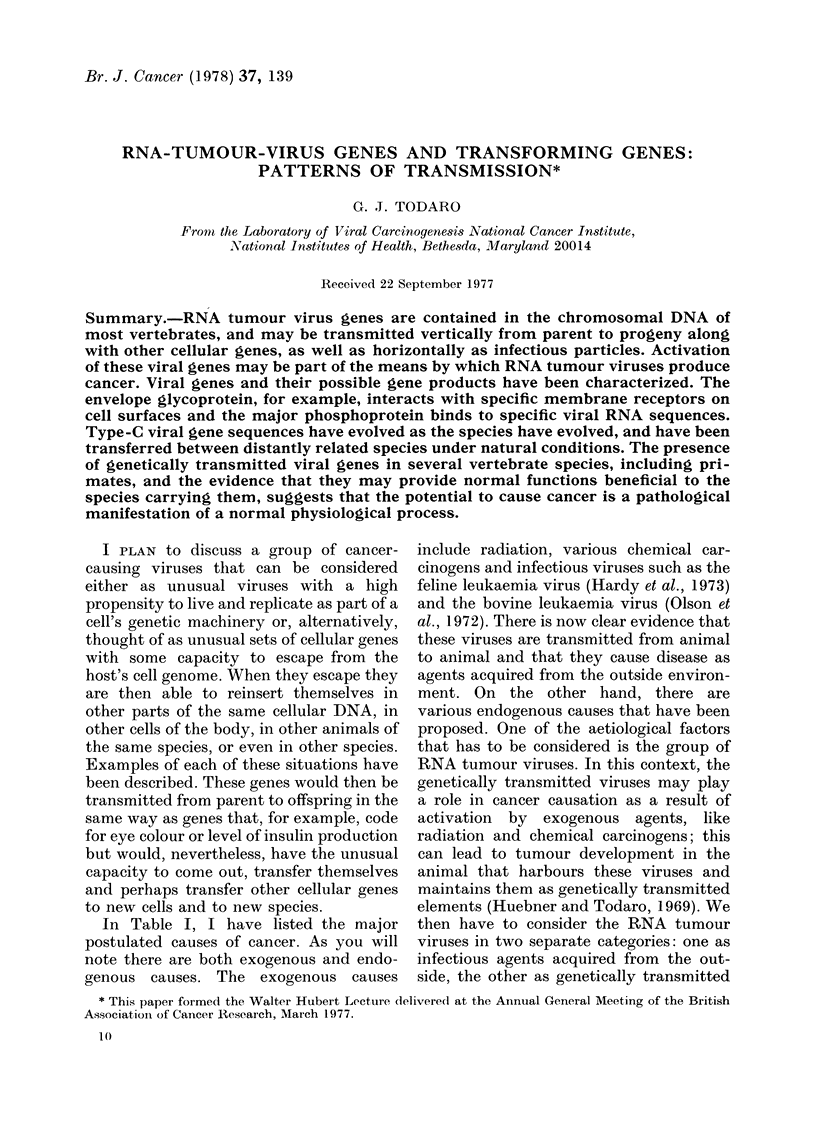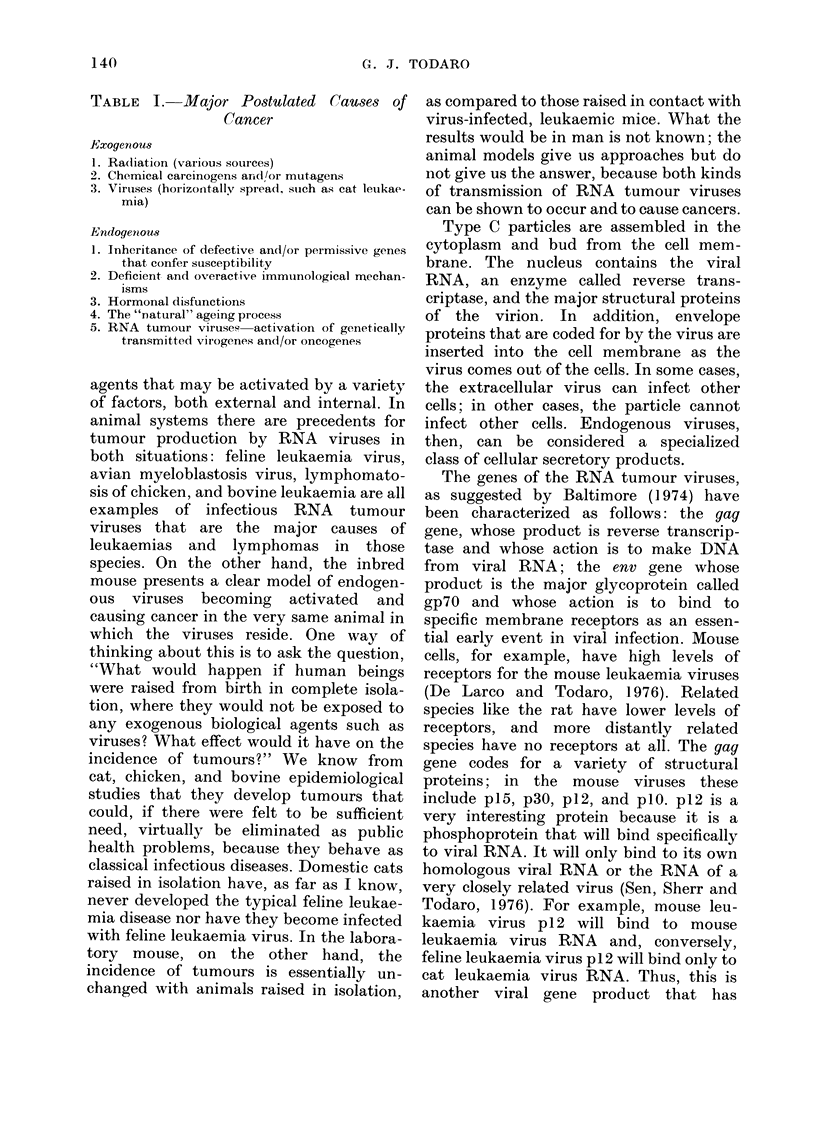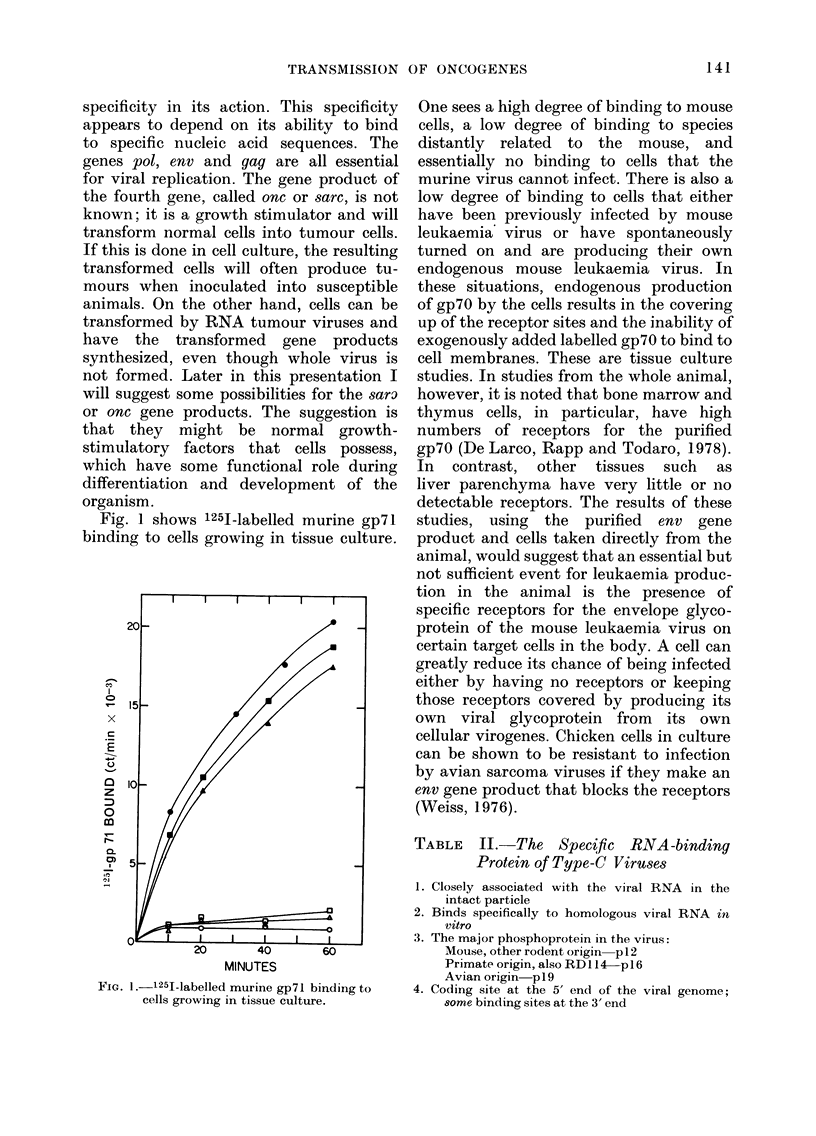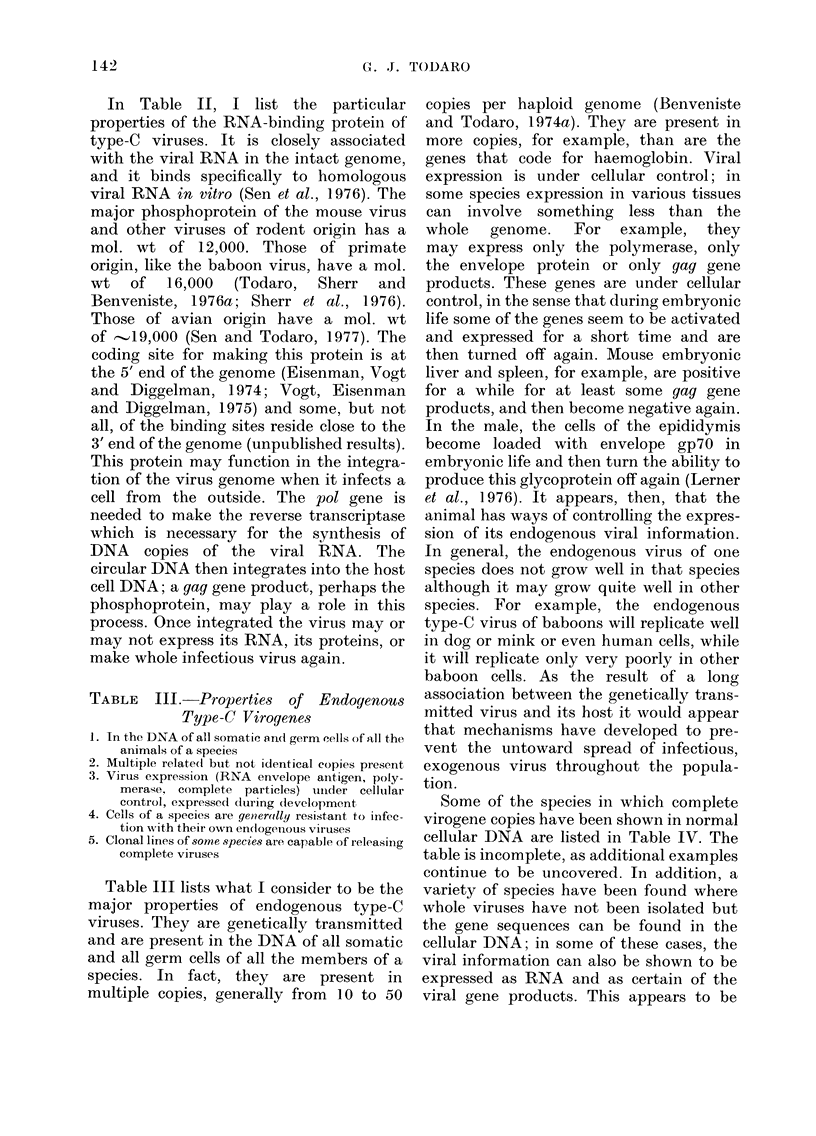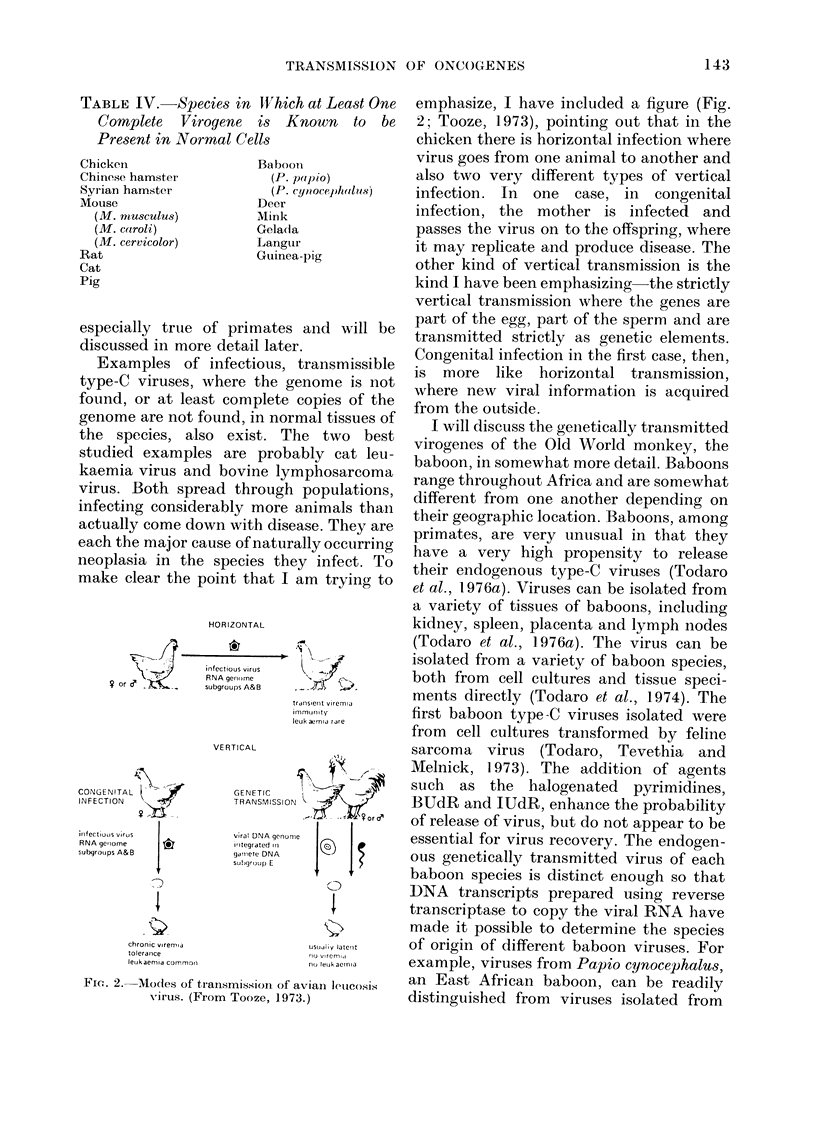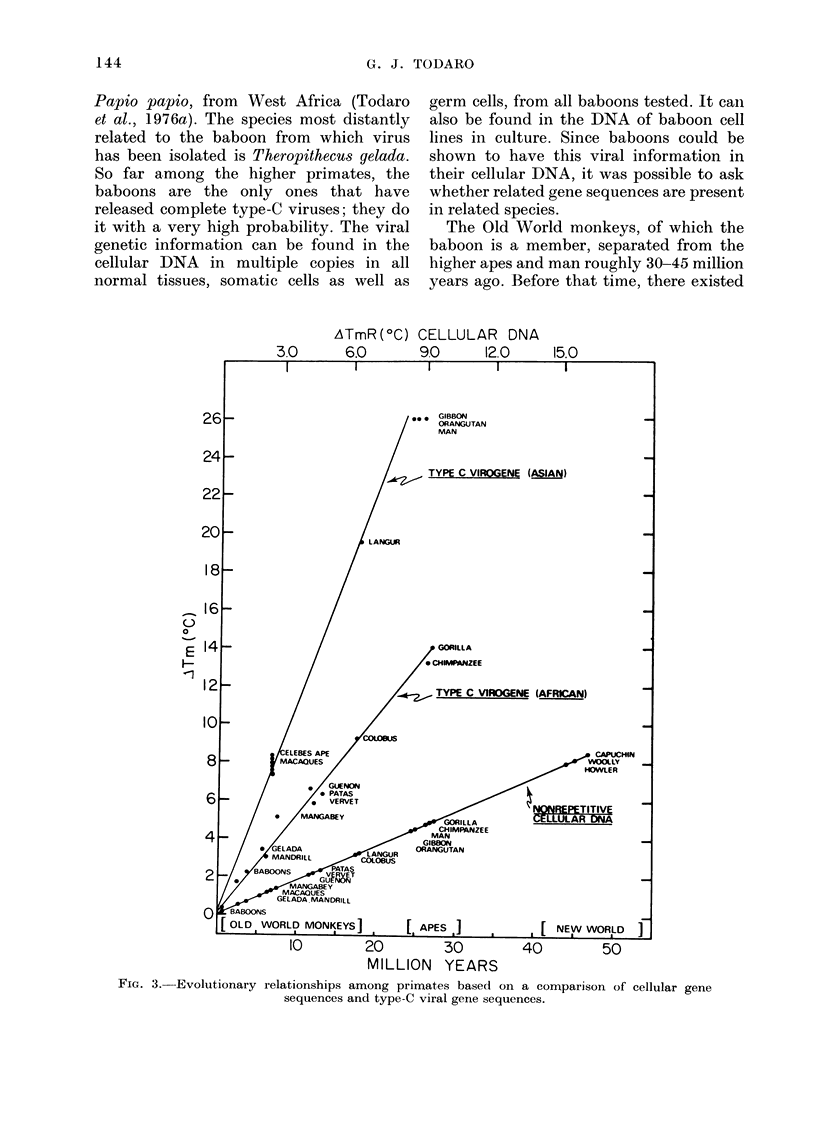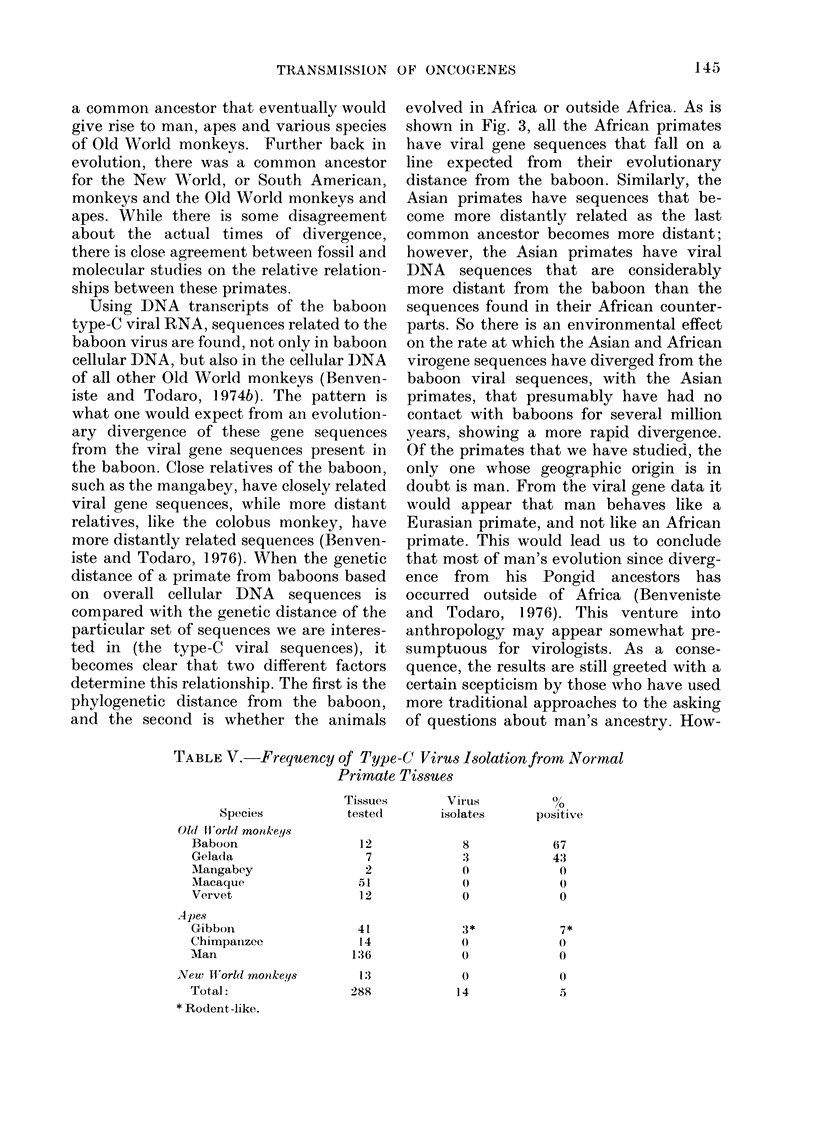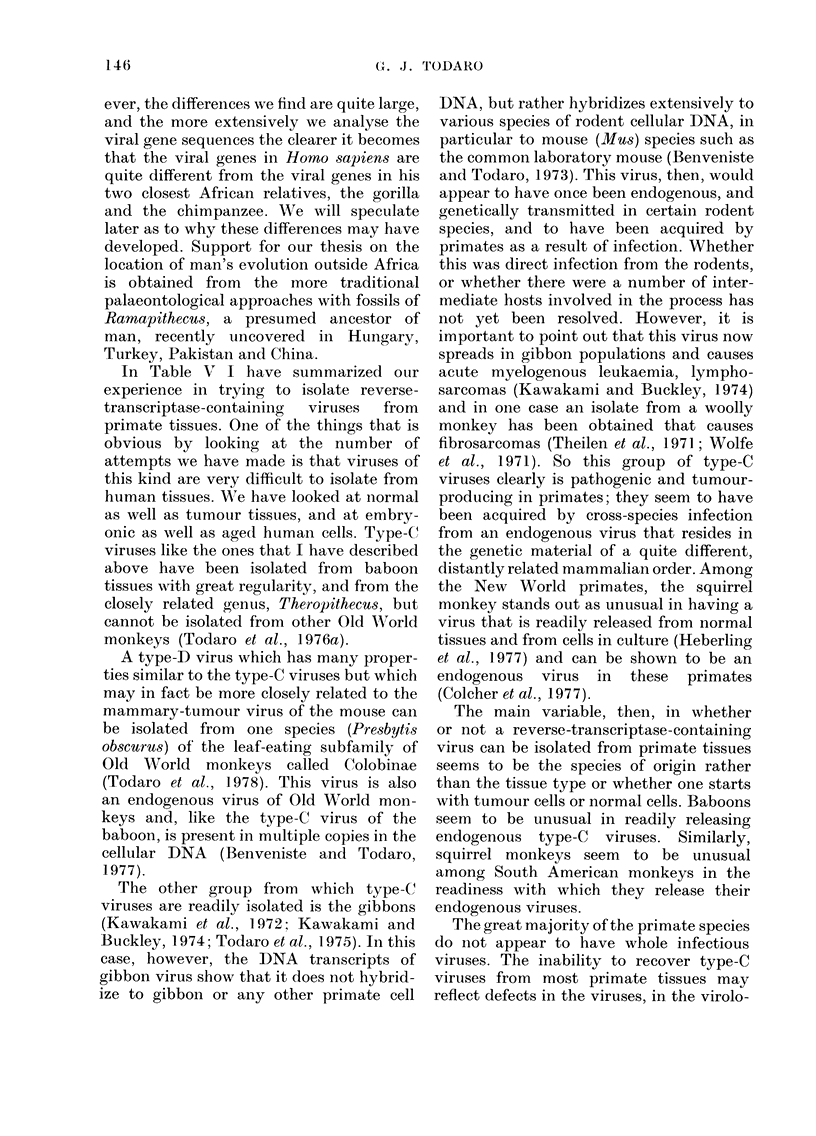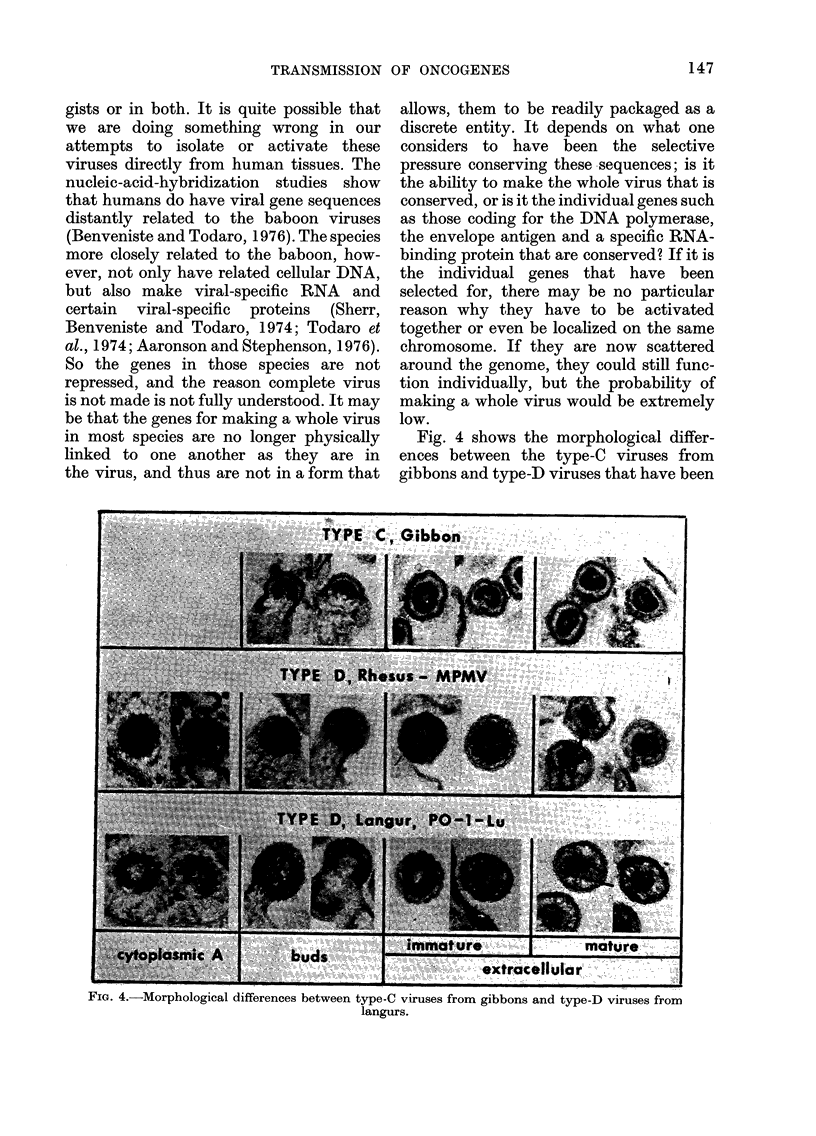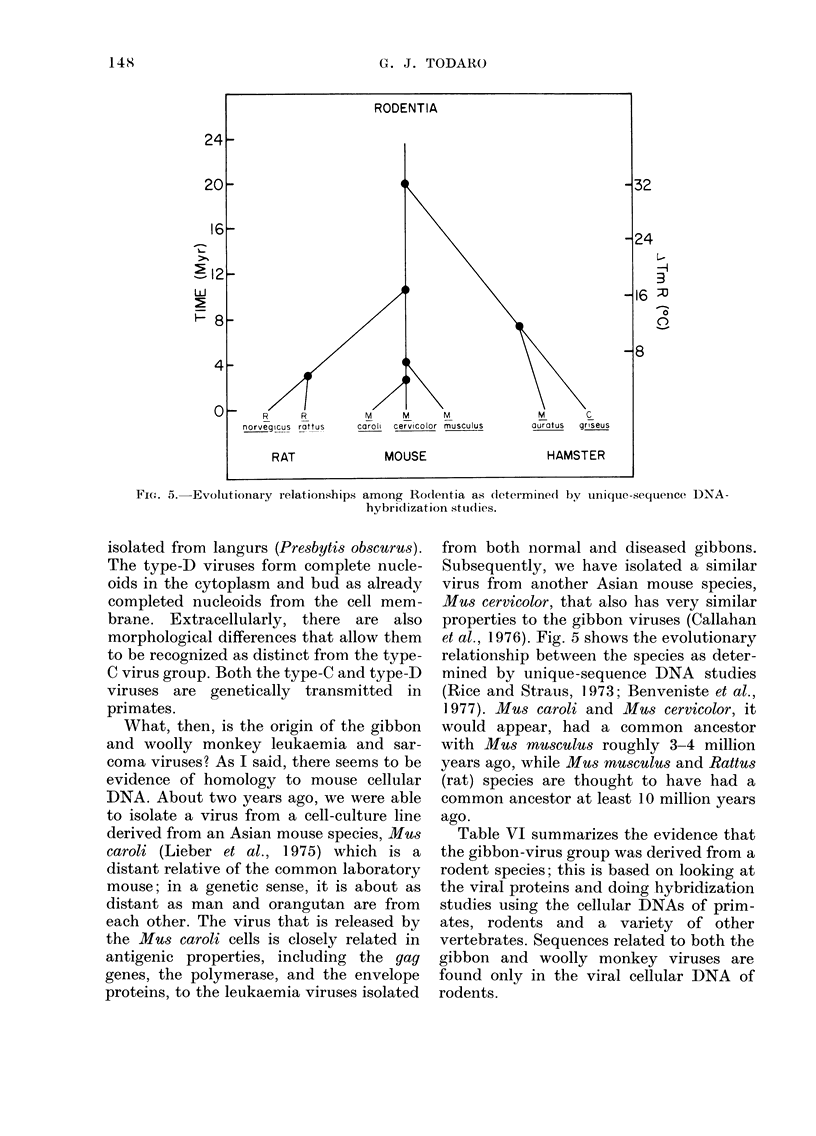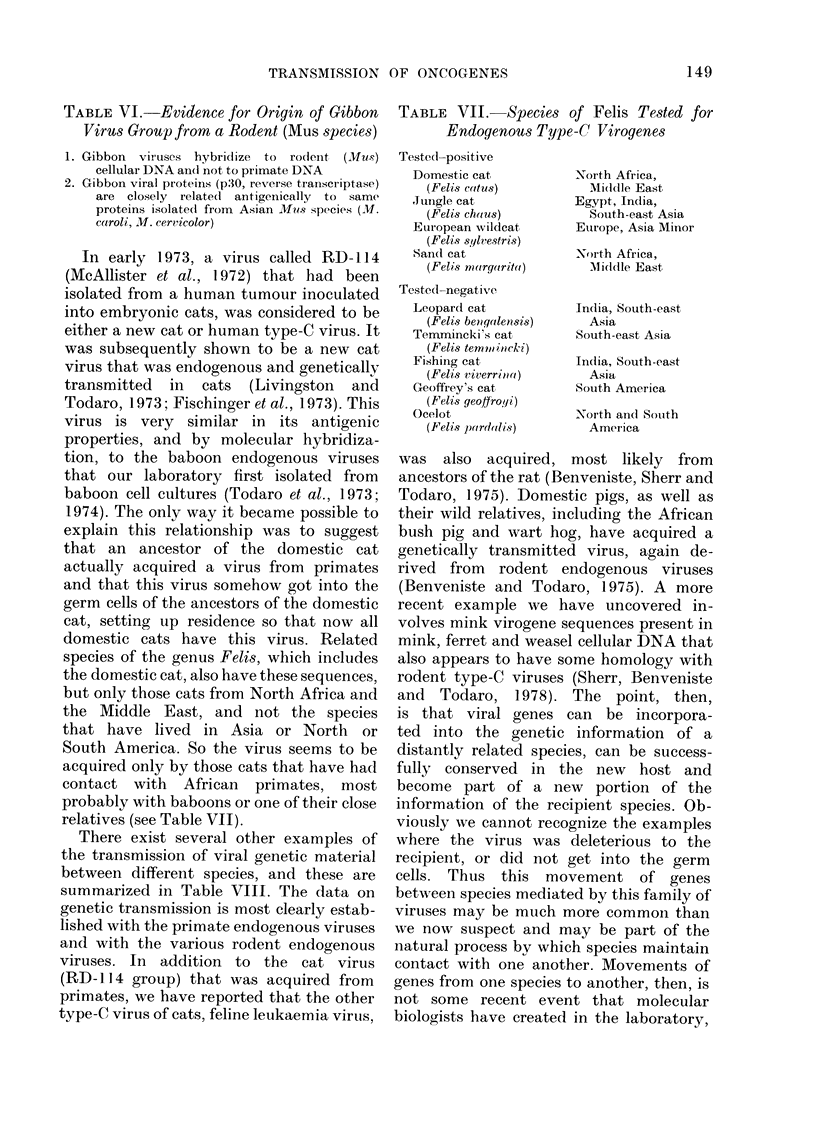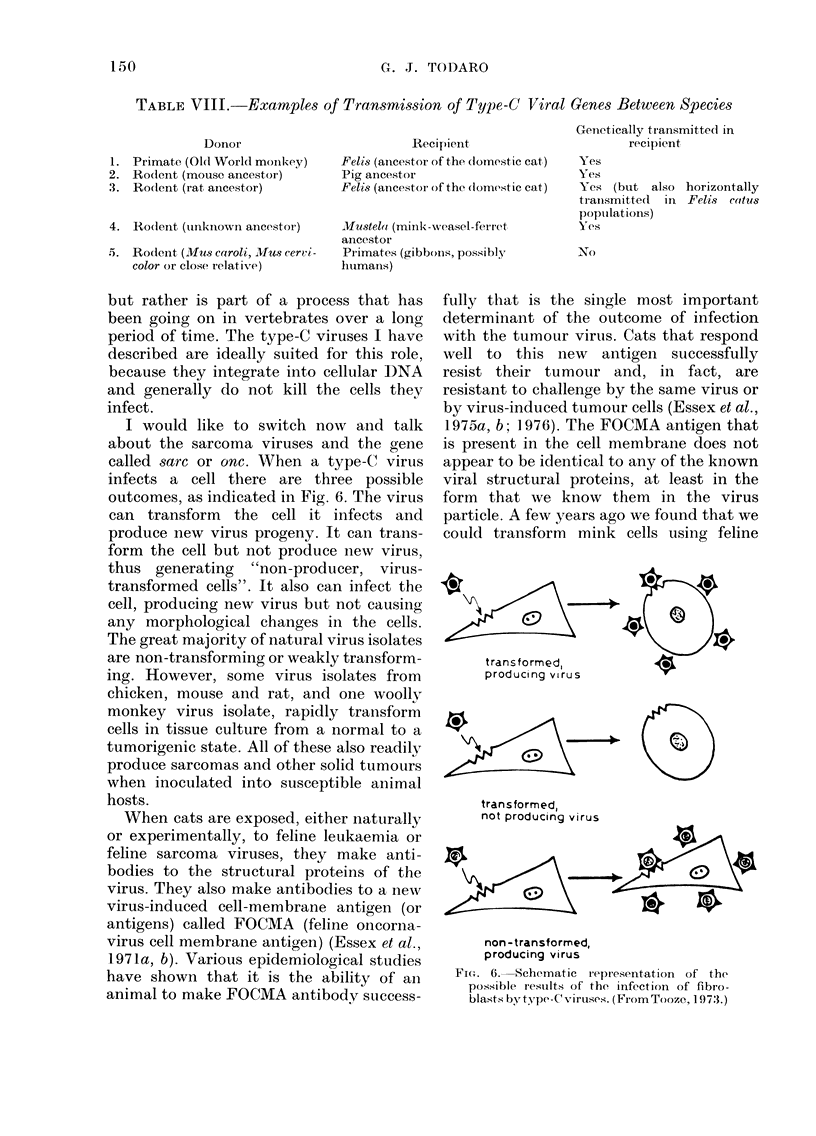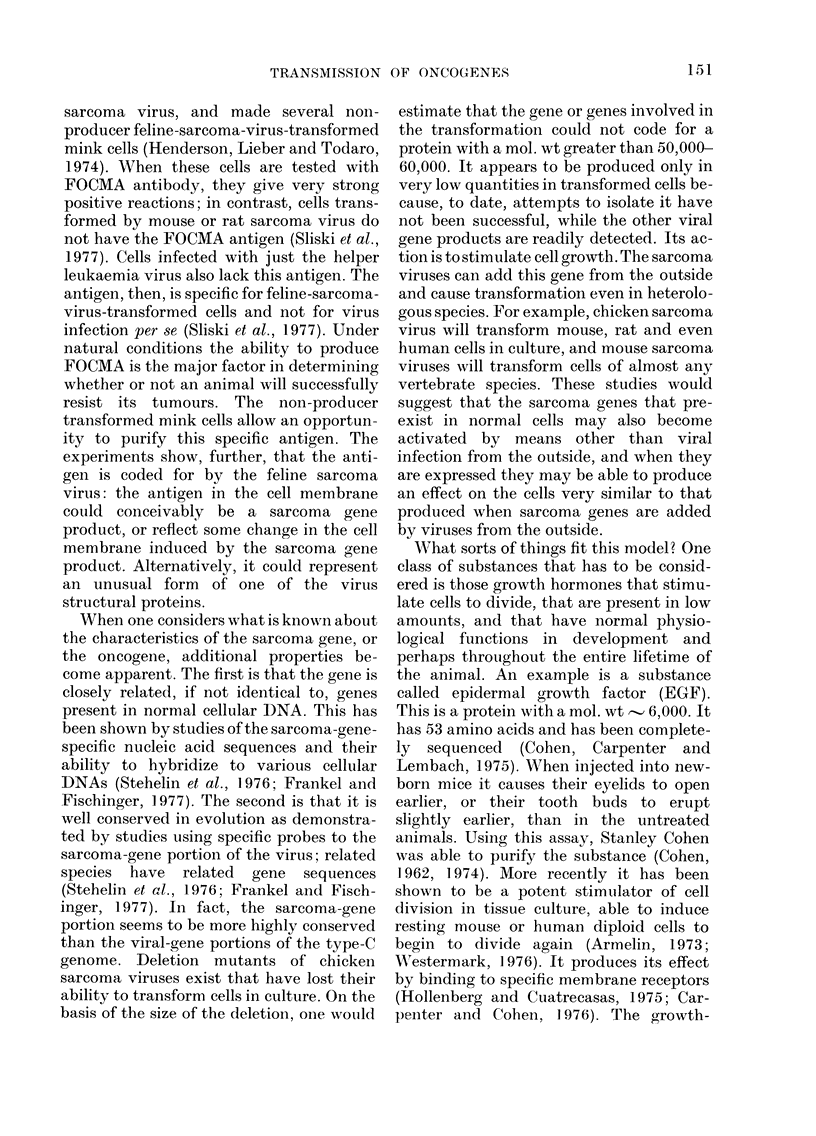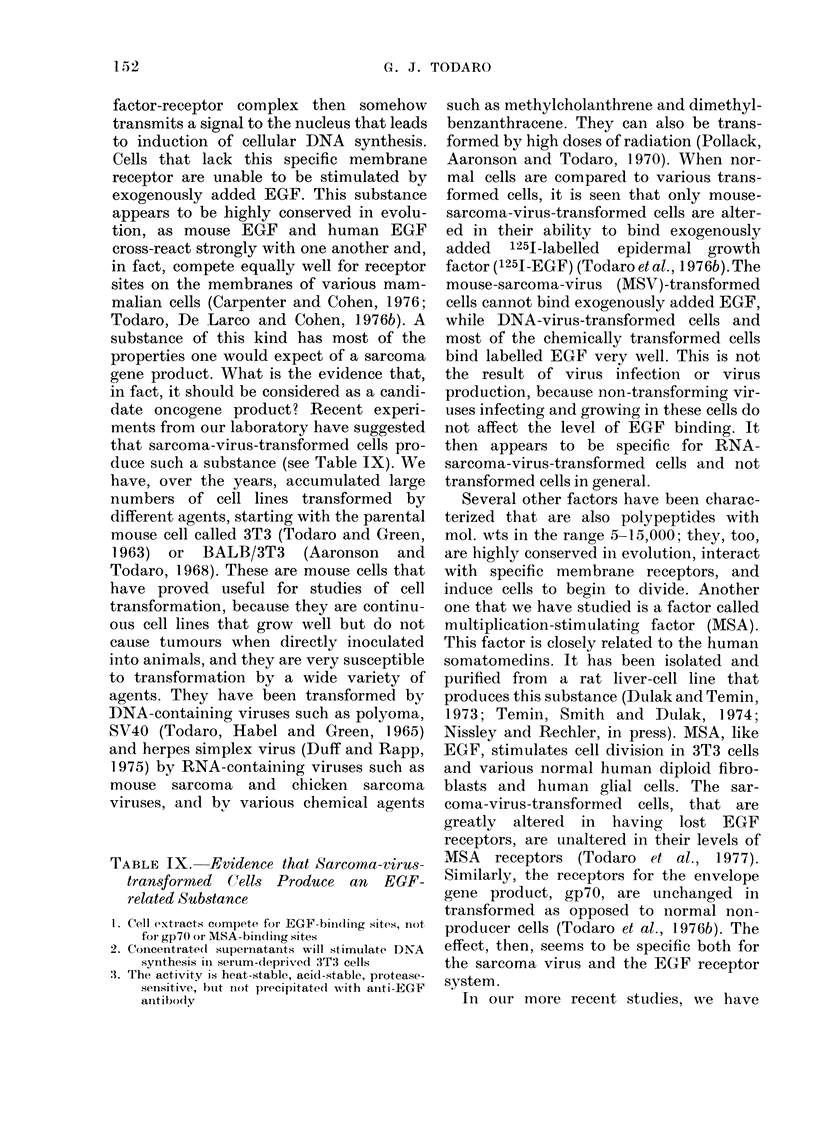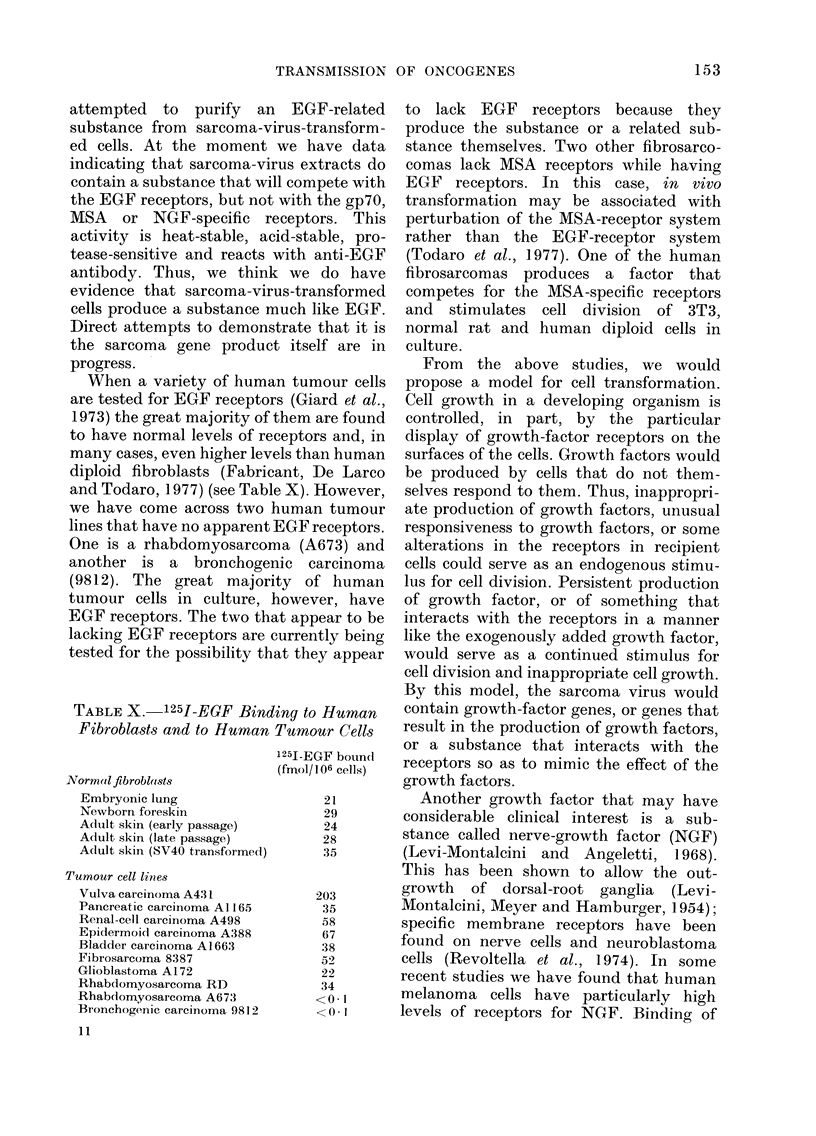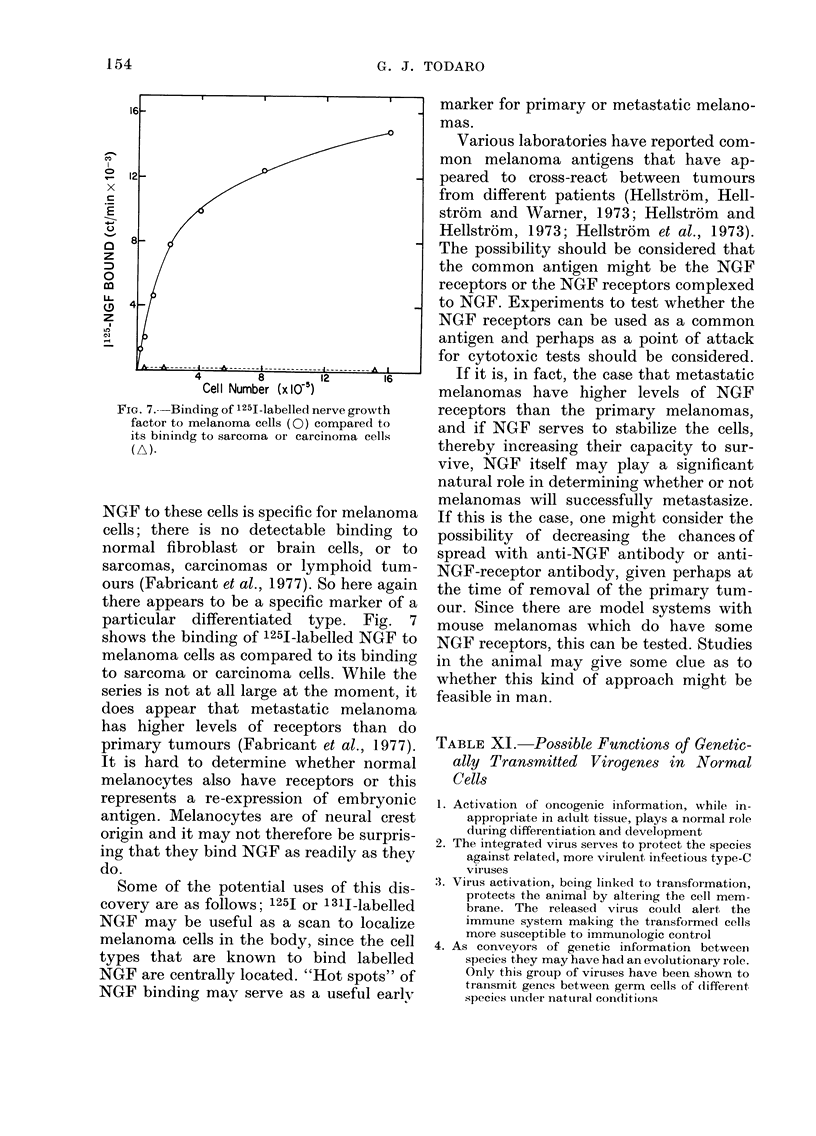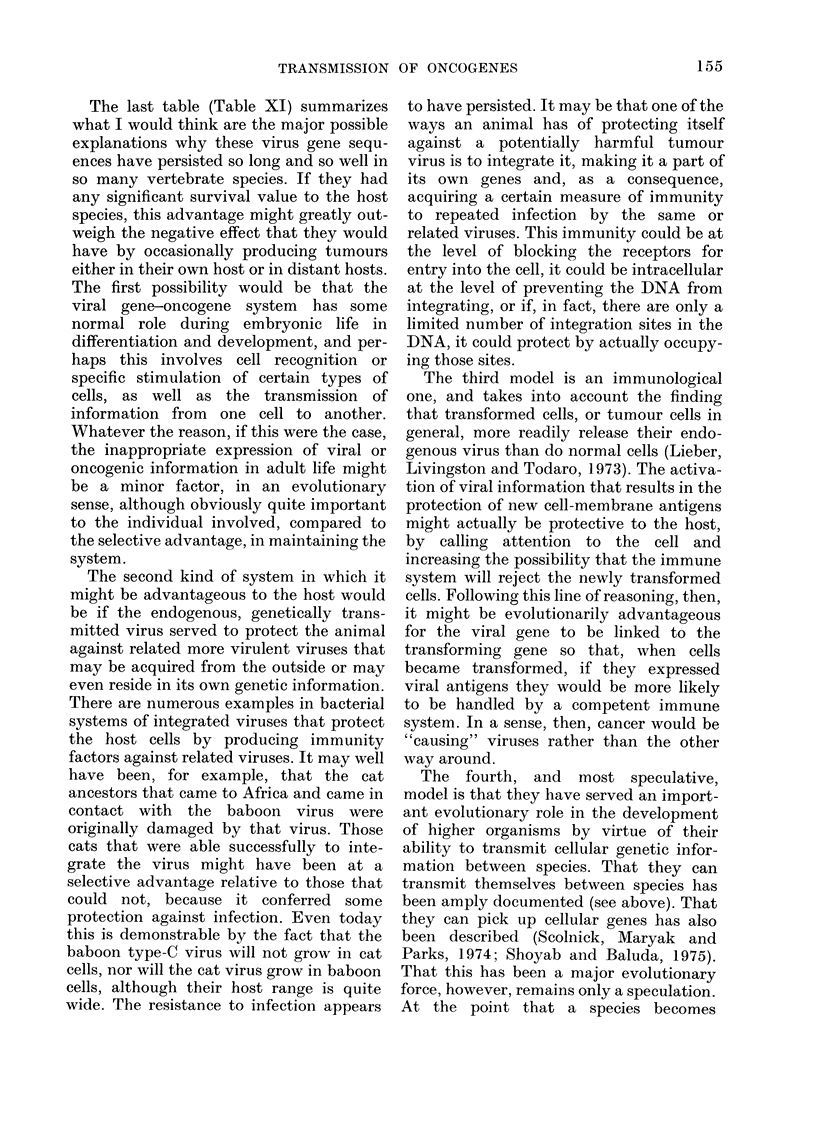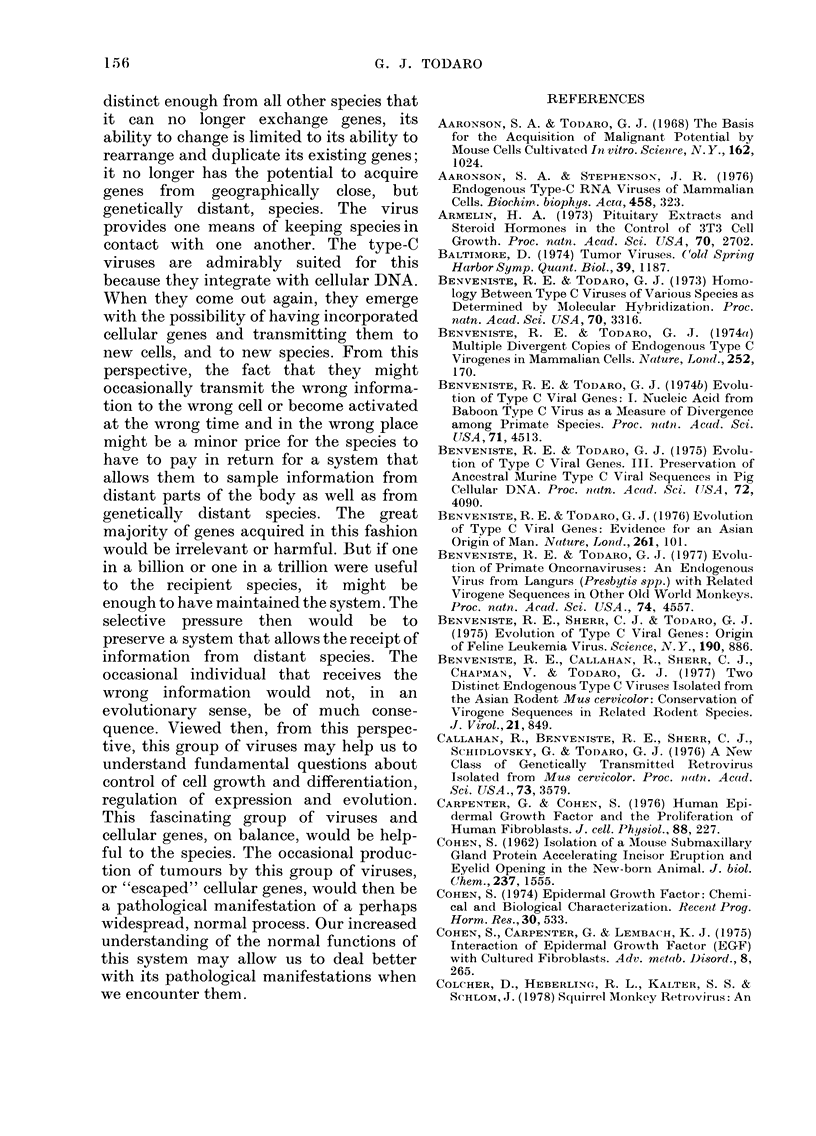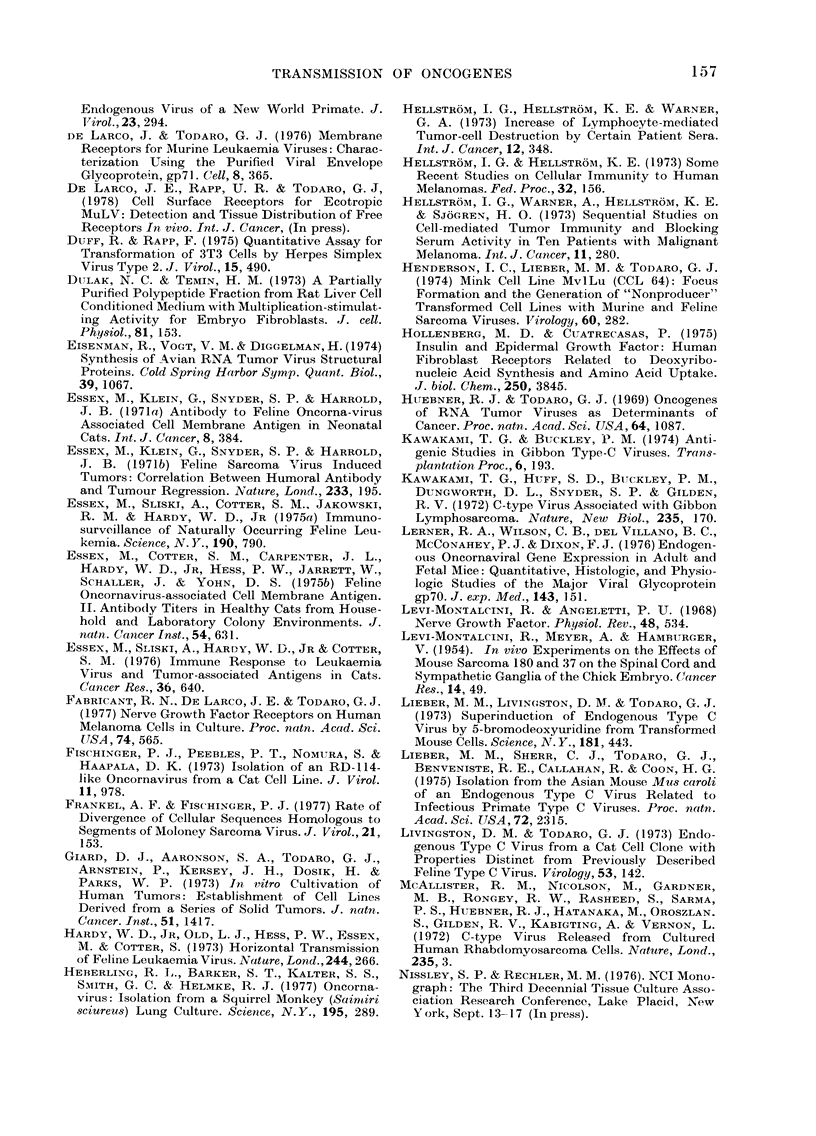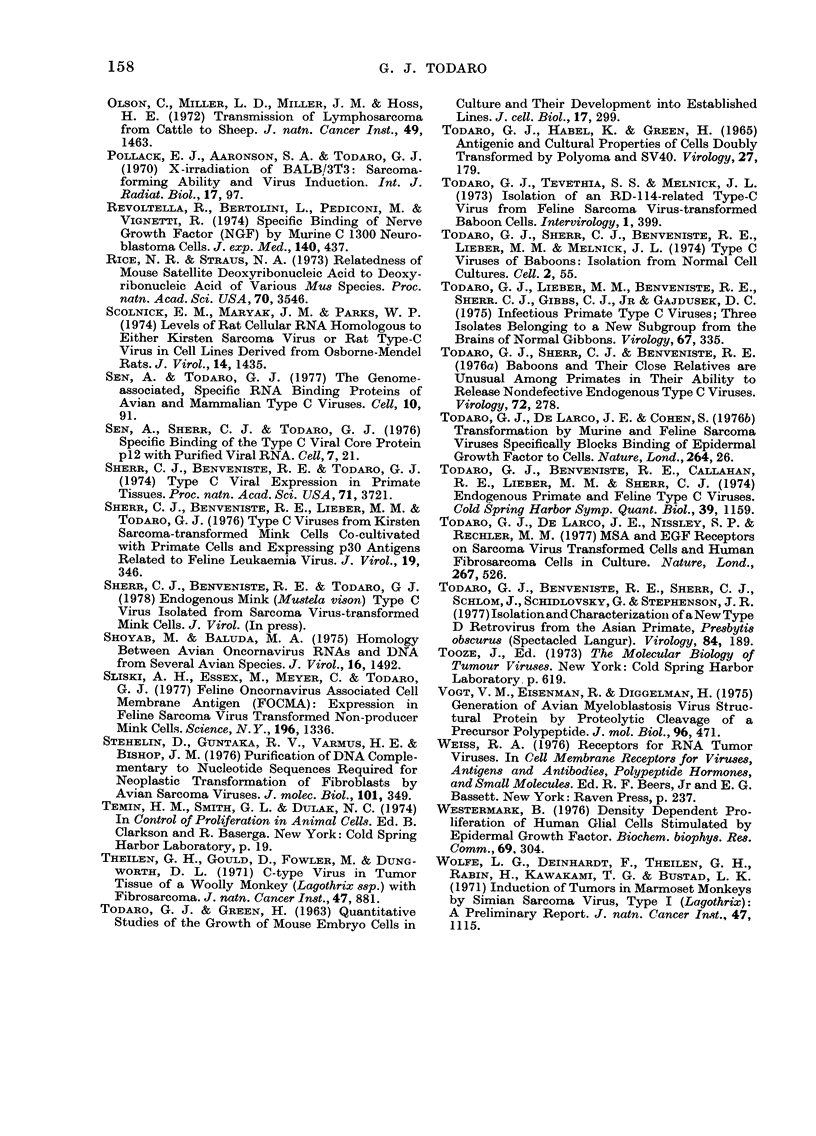# RNA-tumour-virus genes and transforming genes: patterns of transmission.

**DOI:** 10.1038/bjc.1978.23

**Published:** 1978-02

**Authors:** G. J. Todaro

## Abstract

**Images:**


*Br. J. Cancer* (1978) **37**, 139

### RNA-TUMOUR-VIRUS GENES AND TRANSFORMING GENES: PATTERNS OF TRANSMISSION*

G. J. TODARO

*From the Laboratory of Viral Carcinogenesis National Cancer Institute, National Institutes of Health, Bethesda, Maryland* 20014

Received 22 September 1977

**Summary.—RNA tumour virus genes are contained in the chromosomal DNA of most vertebrates, and may be transmitted vertically from parent to progeny along with other cellular genes, as well as horizontally as infectious particles. Activation of these viral genes may be part of the means by which RNA tumour viruses produce cancer. Viral genes and their possible gene products have been characterized. The envelope glycoprotein, for example, interacts with specific membrane receptors on cell surfaces and the major phosphoprotein binds to specific viral RNA sequences. Type-C viral gene sequences have evolved as the species have evolved, and have been transferred between distantly related species under natural conditions. The presence of genetically transmitted viral genes in several vertebrate species, including primates, and the evidence that they may provide normal functions beneficial to the species carrying them, suggests that the potential to cause cancer is a pathological manifestation of a normal physiological process.**

I PLAN to discuss a group of cancer-causing viruses that can be considered either as unusual viruses with a high propensity to live and replicate as part of a cell's genetic machinery or, alternatively, thought of as unusual sets of cellular genes with some capacity to escape from the host's cell genome. When they escape they are then able to reinsert themselves in other parts of the same cellular DNA, in other cells of the body, in other animals of the same species, or even in other species. Examples of each of these situations have been described. These genes would then be transmitted from parent to offspring in the same way as genes that, for example, code for eye colour or level of insulin production but would, nevertheless, have the unusual capacity to come out, transfer themselves and perhaps transfer other cellular genes to new cells and to new species.

In Table I, I have listed the major postulated causes of cancer. As you will note there are both exogenous and endogenous causes. The exogenous causes include radiation, various chemical carcinogens and infectious viruses such as the feline leukaemia virus (Hardy *et al.*, 1973) and the bovine leukaemia virus (Olson *et al.*, 1972). There is now clear evidence that these viruses are transmitted from animal to animal and that they cause disease as agents acquired from the outside environment. On the other hand, there are various endogenous causes that have been proposed. One of the aetiological factors that has to be considered is the group of RNA tumour viruses. In this context, the genetically transmitted viruses may play a role in cancer causation as a result of activation by exogenous agents, like radiation and chemical carcinogens; this can lead to tumour development in the animal that harbours these viruses and maintains them as genetically transmitted elements (Huebner and Todaro, 1969). We then have to consider the RNA tumour viruses in two separate categories: one as infectious agents acquired from the outside, the other as genetically transmitted

* This paper formed the Walter Hubert Lecture delivered at the Annual General Meeting of the British Association of Cancer Research, March 1977.

TABLE I.—*Major Postulated Causes of Cancer*

*Exogenous*

1. Radiation (various sources)

2. Chemical carcinogens and/or mutagens

3. Viruses (horizontally spread, such as cat leukaemia)

*Endogenous*

1. Inheritance of defective and/or permissive genes that confer susceptibility
2. Deficient and overactive immunological mechanisms
3. Hormonal disfunctions
4. The "natural" ageing process
5. RNA tumour viruses—activation of genetically transmitted virogenes and/or oncogenes agents that may be activated by a variety of factors, both external and internal. In animal systems there are precedents for tumour production by RNA viruses in both situations: feline leukaemia virus, avian myeloblastosis virus, lymphomatosis of chicken, and bovine leukaemia are all examples of infectious RNA tumour viruses that are the major causes of leukaemias and lymphomas in those species. On the other hand, the inbred mouse presents a clear model of endogenous viruses becoming activated and causing cancer in the very same animal in which the viruses reside. One way of thinking about this is to ask the question, "What would happen if human beings were raised from birth in complete isolation, where they would not be exposed to any exogenous biological agents such as viruses? What effect would it have on the incidence of tumours?" We know from cat, chicken, and bovine epidemiological studies that they develop tumours that could, if there were felt to be sufficient need, virtually be eliminated as public health problems, because they behave as classical infectious diseases. Domestic cats raised in isolation have, as far as I know, never developed the typical feline leukaemia disease nor have they become infected with feline leukaemia virus. In the laboratory mouse, on the other hand, the incidence of tumours is essentially unchanged with animals raised in isolation, as compared to those raised in contact with virus-infected, leukaemic mice. What the results would be in man is not known; the animal models give us approaches but do not give us the answer, because both kinds of transmission of RNA tumour viruses can be shown to occur and to cause cancers.

Type C particles are assembled in the cytoplasm and bud from the cell membrane. The nucleus contains the viral RNA, an enzyme called reverse transcriptase, and the major structural proteins of the virion. In addition, envelope proteins that are coded for by the virus are inserted into the cell membrane as the virus comes out of the cells. In some cases, the extracellular virus can infect other cells; in other cases, the particle cannot infect other cells. Endogenous viruses, then, can be considered a specialized class of cellular secretory products.

The genes of the RNA tumour viruses, as suggested by Baltimore (1974) have been characterized as follows: the *gag* gene, whose product is reverse transcriptase and whose action is to make DNA from viral RNA; the *env* gene whose product is the major glycoprotein called gp70 and whose action is to bind to specific membrane receptors as an essential early event in viral infection. Mouse cells, for example, have high levels of receptors for the mouse leukaemia viruses (De Larco and Todaro, 1976). Related species like the rat have lower levels of receptors, and more distantly related species have no receptors at all. The *gag* gene codes for a variety of structural proteins; in the mouse viruses these include p15, p30, p12, and p10. p12 is a very interesting protein because it is a phosphoprotein that will bind specifically to viral RNA. It will only bind to its own homologous viral RNA or the RNA of a very closely related virus (Sen, Sherr and Todaro, 1976). For example, mouse leukaemia virus p12 will bind to mouse leukaemia virus RNA and, conversely, feline leukaemia virus p12 will bind only to cat leukaemia virus RNA. Thus, this is another viral gene product that has specificity in its action. This specificity appears to depend on its ability to bind to specific nucleic acid sequences. The genes *pol*, *env* and *gag* are all essential for viral replication. The gene product of the fourth gene, called *onc* or *sarc*, is not known; it is a growth stimulator and will transform normal cells into tumour cells. If this is done in cell culture, the resulting transformed cells will often produce tumours when inoculated into susceptible animals. On the other hand, cells can be transformed by RNA tumour viruses and have the transformed gene products synthesized, even though whole virus is not formed. Later in this presentation I will suggest some possibilities for the *sarc* or *onc* gene products. The suggestion is that they might be normal growth-stimulatory factors that cells possess, which have some functional role during differentiation and development of the organism.

Fig. 1 shows $^{125}$I-labelled murine gp71 binding to cells growing in tissue culture.

FIG. 1.—$^{125}$I-labelled murine gp71 binding to cells growing in tissue culture.

One sees a high degree of binding to mouse cells, a low degree of binding to species distantly related to the mouse, and essentially no binding to cells that the murine virus cannot infect. There is also a low degree of binding to cells that either have been previously infected by mouse leukaemia virus or have spontaneously turned on and are producing their own endogenous mouse leukaemia virus. In these situations, endogenous production of gp70 by the cells results in the covering up of the receptor sites and the inability of exogenously added labelled gp70 to bind to cell membranes. These are tissue culture studies. In studies from the whole animal, however, it is noted that bone marrow and thymus cells, in particular, have high numbers of receptors for the purified gp70 (De Larco, Rapp and Todaro, 1978). In contrast, other tissues such as liver parenchyma have very little or no detectable receptors. The results of these studies, using the purified *env* gene product and cells taken directly from the animal, would suggest that an essential but not sufficient event for leukaemia production in the animal is the presence of specific receptors for the envelope glycoprotein of the mouse leukaemia virus on certain target cells in the body. A cell can greatly reduce its chance of being infected either by having no receptors or keeping those receptors covered by producing its own viral glycoprotein from its own cellular virogenes. Chicken cells in culture can be shown to be resistant to infection by avian sarcoma viruses if they make an *env* gene product that blocks the receptors (Weiss, 1976).

TABLE II.—*The Specific RNA-binding Protein of Type-C Viruses*

1. Closely associated with the viral RNA in the intact particle
2. Binds specifically to homologous viral RNA *in vitro*
3. The major phosphoprotein in the virus:
   Mouse, other rodent origin—p12
   Primate origin, also RD114—p16
   Avian origin—p19
4. Coding site at the 5′ end of the viral genome; *some* binding sites at the 3′ end

In Table II, I list the particular properties of the RNA-binding protein of type-C viruses. It is closely associated with the viral RNA in the intact genome, and it binds specifically to homologous viral RNA *in vitro* (Sen *et al.*, 1976). The major phosphoprotein of the mouse virus and other viruses of rodent origin has a mol. wt of 12,000. Those of primate origin, like the baboon virus, have a mol. wt of 16,000 (Todaro, Sherr and Benveniste, 1976*a*; Sherr *et al.*, 1976). Those of avian origin have a mol. wt of ~19,000 (Sen and Todaro, 1977). The coding site for making this protein is at the 5′ end of the genome (Eisenman, Vogt and Diggelman, 1974; Vogt, Eisenman and Diggelman, 1975) and some, but not all, of the binding sites reside close to the 3′ end of the genome (unpublished results). This protein may function in the integration of the virus genome when it infects a cell from the outside. The *pol* gene is needed to make the reverse transcriptase which is necessary for the synthesis of DNA copies of the viral RNA. The circular DNA then integrates into the host cell DNA; a *gag* gene product, perhaps the phosphoprotein, may play a role in this process. Once integrated the virus may or may not express its RNA, its proteins, or make whole infectious virus again.

Table III.—*Properties of Endogenous Type-C Virogenes*

1. In the DNA of all somatic and germ cells of all the animals of a species
2. Multiple related but not identical copies present
3. Virus expression (RNA envelope antigen, polymerase, complete particles) under cellular control, expressed during development
4. Cells of a species are *generally* resistant to infection with their own endogenous viruses
5. Clonal lines of *some species* are capable of releasing complete viruses

Table III lists what I consider to be the major properties of endogenous type-C viruses. They are genetically transmitted and are present in the DNA of all somatic and all germ cells of all the members of a species. In fact, they are present in multiple copies, generally from 10 to 50 copies per haploid genome (Benveniste and Todaro, 1974*a*). They are present in more copies, for example, than are the genes that code for haemoglobin. Viral expression is under cellular control; in some species expression in various tissues can involve something less than the whole genome. For example, they may express only the polymerase, only the envelope protein or only *gag* gene products. These genes are under cellular control, in the sense that during embryonic life some of the genes seem to be activated and expressed for a short time and are then turned off again. Mouse embryonic liver and spleen, for example, are positive for a while for at least some *gag* gene products, and then become negative again. In the male, the cells of the epididymis become loaded with envelope gp70 in embryonic life and then turn the ability to produce this glycoprotein off again (Lerner *et al.*, 1976). It appears, then, that the animal has ways of controlling the expression of its endogenous viral information. In general, the endogenous virus of one species does not grow well in that species although it may grow quite well in other species. For example, the endogenous type-C virus of baboons will replicate well in dog or mink or even human cells, while it will replicate only very poorly in other baboon cells. As the result of a long association between the genetically transmitted virus and its host it would appear that mechanisms have developed to prevent the untoward spread of infectious, exogenous virus throughout the population.

Some of the species in which complete virogene copies have been shown in normal cellular DNA are listed in Table IV. The table is incomplete, as additional examples continue to be uncovered. In addition, a variety of species have been found where whole viruses have not been isolated but the gene sequences can be found in the cellular DNA; in some of these cases, the viral information can also be shown to be expressed as RNA and as certain of the viral gene products. This appears to be

TABLE IV.—*Species in Which at Least One Complete Virogene is Known to be Present in Normal Cells*

| | |
|---|---|
| Chicken | Baboon |
| Chinese hamster | (*P. papio*) |
| Syrian hamster | (*P. cynocephalus*) |
| Mouse | Deer |
| (*M. musculus*) | Mink |
| (*M. caroli*) | Gelada |
| (*M. cervicolor*) | Langur |
| Rat | Guinea-pig |
| Cat | |
| Pig | |

especially true of primates and will be discussed in more detail later.

Examples of infectious, transmissible type-C viruses, where the genome is not found, or at least complete copies of the genome are not found, in normal tissues of the species, also exist. The two best studied examples are probably cat leukaemia virus and bovine lymphosarcoma virus. Both spread through populations, infecting considerably more animals than actually come down with disease. They are each the major cause of naturally occurring neoplasia in the species they infect. To make clear the point that I am trying to emphasize, I have included a figure (Fig. 2; Tooze, 1973), pointing out that in the chicken there is horizontal infection where virus goes from one animal to another and also two very different types of vertical infection. In one case, in congenital infection, the mother is infected and passes the virus on to the offspring, where it may replicate and produce disease. The other kind of vertical transmission is the kind I have been emphasizing—the strictly vertical transmission where the genes are part of the egg, part of the sperm and are transmitted strictly as genetic elements. Congenital infection in the first case, then, is more like horizontal transmission, where new viral information is acquired from the outside.

FIG. 2.—Modes of transmission of avian leucosis virus. (From Tooze, 1973.)

I will discuss the genetically transmitted virogenes of the Old World monkey, the baboon, in somewhat more detail. Baboons range throughout Africa and are somewhat different from one another depending on their geographic location. Baboons, among primates, are very unusual in that they have a very high propensity to release their endogenous type-C viruses (Todaro *et al.*, 1976*a*). Viruses can be isolated from a variety of tissues of baboons, including kidney, spleen, placenta and lymph nodes (Todaro *et al.*, 1976*a*). The virus can be isolated from a variety of baboon species, both from cell cultures and tissue specimens directly (Todaro *et al.*, 1974). The first baboon type-C viruses isolated were from cell cultures transformed by feline sarcoma virus (Todaro, Tevethia and Melnick, 1973). The addition of agents such as the halogenated pyrimidines, BUdR and IUdR, enhance the probability of release of virus, but do not appear to be essential for virus recovery. The endogenous genetically transmitted virus of each baboon species is distinct enough so that DNA transcripts prepared using reverse transcriptase to copy the viral RNA have made it possible to determine the species of origin of different baboon viruses. For example, viruses from *Papio cynocephalus*, an East African baboon, can be readily distinguished from viruses isolated from

*Papio papio*, from West Africa (Todaro *et al.*, 1976*a*). The species most distantly related to the baboon from which virus has been isolated is *Theropithecus gelada*. So far among the higher primates, the baboons are the only ones that have released complete type-C viruses; they do it with a very high probability. The viral genetic information can be found in the cellular DNA in multiple copies in all normal tissues, somatic cells as well as germ cells, from all baboons tested. It can also be found in the DNA of baboon cell lines in culture. Since baboons could be shown to have this viral information in their cellular DNA, it was possible to ask whether related gene sequences are present in related species.

The Old World monkeys, of which the baboon is a member, separated from the higher apes and man roughly 30–45 million years ago. Before that time, there existed

FIG. 3.—Evolutionary relationships among primates based on a comparison of cellular gene sequences and type-C viral gene sequences.

a common ancestor that eventually would give rise to man, apes and various species of Old World monkeys. Further back in evolution, there was a common ancestor for the New World, or South American, monkeys and the Old World monkeys and apes. While there is some disagreement about the actual times of divergence, there is close agreement between fossil and molecular studies on the relative relationships between these primates.

Using DNA transcripts of the baboon type-C viral RNA, sequences related to the baboon virus are found, not only in baboon cellular DNA, but also in the cellular DNA of all other Old World monkeys (Benveniste and Todaro, 1974*b*). The pattern is what one would expect from an evolutionary divergence of these gene sequences from the viral gene sequences present in the baboon. Close relatives of the baboon, such as the mangabey, have closely related viral gene sequences, while more distant relatives, like the colobus monkey, have more distantly related sequences (Benveniste and Todaro, 1976). When the genetic distance of a primate from baboons based on overall cellular DNA sequences is compared with the genetic distance of the particular set of sequences we are interested in (the type-C viral sequences), it becomes clear that two different factors determine this relationship. The first is the phylogenetic distance from the baboon, and the second is whether the animals evolved in Africa or outside Africa. As is shown in Fig. 3, all the African primates have viral gene sequences that fall on a line expected from their evolutionary distance from the baboon. Similarly, the Asian primates have sequences that become more distantly related as the last common ancestor becomes more distant; however, the Asian primates have viral DNA sequences that are considerably more distant from the baboon than the sequences found in their African counterparts. So there is an environmental effect on the rate at which the Asian and African virogene sequences have diverged from the baboon viral sequences, with the Asian primates, that presumably have had no contact with baboons for several million years, showing a more rapid divergence. Of the primates that we have studied, the only one whose geographic origin is in doubt is man. From the viral gene data it would appear that man behaves like a Eurasian primate, and not like an African primate. This would lead us to conclude that most of man's evolution since divergence from his Pongid ancestors has occurred outside of Africa (Benveniste and Todaro, 1976). This venture into anthropology may appear somewhat presumptuous for virologists. As a consequence, the results are still greeted with a certain scepticism by those who have used more traditional approaches to the asking of questions about man's ancestry. How-

TABLE V.—*Frequency of Type-C Virus Isolation from Normal Primate Tissues*

| Species | Tissues tested | Virus isolates | % positive |
|---|---|---|---|
| *Old World monkeys* | | | |
| Baboon | 12 | 8 | 67 |
| Gelada | 7 | 3 | 43 |
| Mangabey | 2 | 0 | 0 |
| Macaque | 51 | 0 | 0 |
| Vervet | 12 | 0 | 0 |
| *Apes* | | | |
| Gibbon | 41 | 3* | 7* |
| Chimpanzee | 14 | 0 | 0 |
| Man | 136 | 0 | 0 |
| *New World monkeys* | 13 | 0 | 0 |
| Total: | 288 | 14 | 5 |

* Rodent-like.

ever, the differences we find are quite large, and the more extensively we analyse the viral gene sequences the clearer it becomes that the viral genes in *Homo sapiens* are quite different from the viral genes in his two closest African relatives, the gorilla and the chimpanzee. We will speculate later as to why these differences may have developed. Support for our thesis on the location of man's evolution outside Africa is obtained from the more traditional palaeontological approaches with fossils of *Ramapithecus*, a presumed ancestor of man, recently uncovered in Hungary, Turkey, Pakistan and China.

In Table V I have summarized our experience in trying to isolate reverse-transcriptase-containing viruses from primate tissues. One of the things that is obvious by looking at the number of attempts we have made is that viruses of this kind are very difficult to isolate from human tissues. We have looked at normal as well as tumour tissues, and at embryonic as well as aged human cells. Type-C viruses like the ones that I have described above have been isolated from baboon tissues with great regularity, and from the closely related genus, *Theropithecus*, but cannot be isolated from other Old World monkeys (Todaro *et al.*, 1976*a*).

A type-D virus which has many properties similar to the type-C viruses but which may in fact be more closely related to the mammary-tumour virus of the mouse can be isolated from one species (*Presbytis obscurus*) of the leaf-eating subfamily of Old World monkeys called Colobinae (Todaro *et al.*, 1978). This virus is also an endogenous virus of Old World monkeys and, like the type-C virus of the baboon, is present in multiple copies in the cellular DNA (Benveniste and Todaro, 1977).

The other group from which type-C viruses are readily isolated is the gibbons (Kawakami *et al.*, 1972; Kawakami and Buckley, 1974; Todaro *et al.*, 1975). In this case, however, the DNA transcripts of gibbon virus show that it does not hybridize to gibbon or any other primate cell DNA, but rather hybridizes extensively to various species of rodent cellular DNA, in particular to mouse (*Mus*) species such as the common laboratory mouse (Benveniste and Todaro, 1973). This virus, then, would appear to have once been endogenous, and genetically transmitted in certain rodent species, and to have been acquired by primates as a result of infection. Whether this was direct infection from the rodents, or whether there were a number of intermediate hosts involved in the process has not yet been resolved. However, it is important to point out that this virus now spreads in gibbon populations and causes acute myelogenous leukaemia, lymphosarcomas (Kawakami and Buckley, 1974) and in one case an isolate from a woolly monkey has been obtained that causes fibrosarcomas (Theilen *et al.*, 1971; Wolfe *et al.*, 1971). So this group of type-C viruses clearly is pathogenic and tumour-producing in primates; they seem to have been acquired by cross-species infection from an endogenous virus that resides in the genetic material of a quite different, distantly related mammalian order. Among the New World primates, the squirrel monkey stands out as unusual in having a virus that is readily released from normal tissues and from cells in culture (Heberling *et al.*, 1977) and can be shown to be an endogenous virus in these primates (Colcher *et al.*, 1977).

The main variable, then, in whether or not a reverse-transcriptase-containing virus can be isolated from primate tissues seems to be the species of origin rather than the tissue type or whether one starts with tumour cells or normal cells. Baboons seem to be unusual in readily releasing endogenous type-C viruses. Similarly, squirrel monkeys seem to be unusual among South American monkeys in the readiness with which they release their endogenous viruses.

The great majority of the primate species do not appear to have whole infectious viruses. The inability to recover type-C viruses from most primate tissues may reflect defects in the viruses, in the virologists or in both. It is quite possible that we are doing something wrong in our attempts to isolate or activate these viruses directly from human tissues. The nucleic-acid-hybridization studies show that humans do have viral gene sequences distantly related to the baboon viruses (Benveniste and Todaro, 1976). The species more closely related to the baboon, however, not only have related cellular DNA, but also make viral-specific RNA and certain viral-specific proteins (Sherr, Benveniste and Todaro, 1974; Todaro *et al.*, 1974; Aaronson and Stephenson, 1976). So the genes in those species are not repressed, and the reason complete virus is not made is not fully understood. It may be that the genes for making a whole virus in most species are no longer physically linked to one another as they are in the virus, and thus are not in a form that allows, them to be readily packaged as a discrete entity. It depends on what one considers to have been the selective pressure conserving these sequences; is it the ability to make the whole virus that is conserved, or is it the individual genes such as those coding for the DNA polymerase, the envelope antigen and a specific RNA-binding protein that are conserved? If it is the individual genes that have been selected for, there may be no particular reason why they have to be activated together or even be localized on the same chromosome. If they are now scattered around the genome, they could still function individually, but the probability of making a whole virus would be extremely low.

Fig. 4 shows the morphological differences between the type-C viruses from gibbons and type-D viruses that have been

FIG. 4.—Morphological differences between type-C viruses from gibbons and type-D viruses from langurs.

FIG. 5.—Evolutionary relationships among Rodentia as determined by unique-sequence DNA-hybridization studies.

isolated from langurs (*Presbytis obscurus*). The type-D viruses form complete nucleoids in the cytoplasm and bud as already completed nucleoids from the cell membrane. Extracellularly, there are also morphological differences that allow them to be recognized as distinct from the type-C virus group. Both the type-C and type-D viruses are genetically transmitted in primates.

What, then, is the origin of the gibbon and woolly monkey leukaemia and sarcoma viruses? As I said, there seems to be evidence of homology to mouse cellular DNA. About two years ago, we were able to isolate a virus from a cell-culture line derived from an Asian mouse species, *Mus caroli* (Lieber *et al.*, 1975) which is a distant relative of the common laboratory mouse; in a genetic sense, it is about as distant as man and orangutan are from each other. The virus that is released by the *Mus caroli* cells is closely related in antigenic properties, including the *gag* genes, the polymerase, and the envelope proteins, to the leukaemia viruses isolated from both normal and diseased gibbons. Subsequently, we have isolated a similar virus from another Asian mouse species, *Mus cervicolor*, that also has very similar properties to the gibbon viruses (Callahan *et al.*, 1976). Fig. 5 shows the evolutionary relationship between the species as determined by unique-sequence DNA studies (Rice and Straus, 1973; Benveniste *et al.*, 1977). *Mus caroli* and *Mus cervicolor*, it would appear, had a common ancestor with *Mus musculus* roughly 3–4 million years ago, while *Mus musculus* and *Rattus* (rat) species are thought to have had a common ancestor at least 10 million years ago.

Table VI summarizes the evidence that the gibbon-virus group was derived from a rodent species; this is based on looking at the viral proteins and doing hybridization studies using the cellular DNAs of primates, rodents and a variety of other vertebrates. Sequences related to both the gibbon and woolly monkey viruses are found only in the viral cellular DNA of rodents.

TABLE VI.—*Evidence for Origin of Gibbon Virus Group from a Rodent* (Mus *species*)

1. Gibbon viruses hybridize to rodent (*Mus*) cellular DNA and not to primate DNA
2. Gibbon viral proteins (p30, reverse transcriptase) are closely related antigenically to same proteins isolated from Asian *Mus* species (*M. caroli*, *M. cervicolor*)

In early 1973, a virus called RD-114 (McAllister *et al.*, 1972) that had been isolated from a human tumour inoculated into embryonic cats, was considered to be either a new cat or human type-C virus. It was subsequently shown to be a new cat virus that was endogenous and genetically transmitted in cats (Livingston and Todaro, 1973; Fischinger *et al.*, 1973). This virus is very similar in its antigenic properties, and by molecular hybridization, to the baboon endogenous viruses that our laboratory first isolated from baboon cell cultures (Todaro *et al.*, 1973; 1974). The only way it became possible to explain this relationship was to suggest that an ancestor of the domestic cat actually acquired a virus from primates and that this virus somehow got into the germ cells of the ancestors of the domestic cat, setting up residence so that now all domestic cats have this virus. Related species of the genus *Felis*, which includes the domestic cat, also have these sequences, but only those cats from North Africa and the Middle East, and not the species that have lived in Asia or North or South America. So the virus seems to be acquired only by those cats that have had contact with African primates, most probably with baboons or one of their close relatives (see Table VII).

There exist several other examples of the transmission of viral genetic material between different species, and these are summarized in Table VIII. The data on genetic transmission is most clearly established with the primate endogenous viruses and with the various rodent endogenous viruses. In addition to the cat virus (RD-114 group) that was acquired from primates, we have reported that the other type-C virus of cats, feline leukaemia virus,

TABLE VII.—*Species of* Felis *Tested for Endogenous Type-C Virogenes*

| | |
|---|---|
| Tested-positive | |
| Domestic cat (*Felis catus*) | North Africa, Middle East |
| Jungle cat (*Felis chaus*) | Egypt, India, South-east Asia |
| European wildcat (*Felis sylvestris*) | Europe, Asia Minor |
| Sand cat (*Felis margarita*) | North Africa, Middle East |
| Tested-negative | |
| Leopard cat (*Felis bengalensis*) | India, South-east Asia |
| Temmincki's cat (*Felis temmincki*) | South-east Asia |
| Fishing cat (*Felis viverrina*) | India, South-east Asia |
| Geoffrey's cat (*Felis geoffroyi*) | South America |
| Ocelot (*Felis pardalis*) | North and South America |

was also acquired, most likely from ancestors of the rat (Benveniste, Sherr and Todaro, 1975). Domestic pigs, as well as their wild relatives, including the African bush pig and wart hog, have acquired a genetically transmitted virus, again derived from rodent endogenous viruses (Benveniste and Todaro, 1975). A more recent example we have uncovered involves mink virogene sequences present in mink, ferret and weasel cellular DNA that also appears to have some homology with rodent type-C viruses (Sherr, Benveniste and Todaro, 1978). The point, then, is that viral genes can be incorporated into the genetic information of a distantly related species, can be successfully conserved in the new host and become part of a new portion of the information of the recipient species. Obviously we cannot recognize the examples where the virus was deleterious to the recipient, or did not get into the germ cells. Thus this movement of genes between species mediated by this family of viruses may be much more common than we now suspect and may be part of the natural process by which species maintain contact with one another. Movements of genes from one species to another, then, is not some recent event that molecular biologists have created in the laboratory,

TABLE VIII.—*Examples of Transmission of Type-C Viral Genes Between Species*

| | Donor | Recipient | Genetically transmitted in recipient |
|---|---|---|---|
| 1. | Primate (Old World monkey) | *Felis* (ancestor of the domestic cat) | Yes |
| 2. | Rodent (mouse ancestor) | Pig ancestor | Yes |
| 3. | Rodent (rat ancestor) | *Felis* (ancestor of the domestic cat) | Yes (but also horizontally transmitted in *Felis catus* populations) |
| 4. | Rodent (unknown ancestor) | *Mustela* (mink-weasel-ferret ancestor | Yes |
| 5. | Rodent (*Mus caroli, Mus cervicolor* or close relative) | Primates (gibbons, possibly humans) | No |

but rather is part of a process that has been going on in vertebrates over a long period of time. The type-C viruses I have described are ideally suited for this role, because they integrate into cellular DNA and generally do not kill the cells they infect.

I would like to switch now and talk about the sarcoma viruses and the gene called *sarc* or *onc*. When a type-C virus infects a cell there are three possible outcomes, as indicated in Fig. 6. The virus can transform the cell it infects and produce new virus progeny. It can transform the cell but not produce new virus, thus generating "non-producer, virus-transformed cells". It also can infect the cell, producing new virus but not causing any morphological changes in the cells. The great majority of natural virus isolates are non-transforming or weakly transforming. However, some virus isolates from chicken, mouse and rat, and one woolly monkey virus isolate, rapidly transform cells in tissue culture from a normal to a tumorigenic state. All of these also readily produce sarcomas and other solid tumours when inoculated into susceptible animal hosts.

When cats are exposed, either naturally or experimentally, to feline leukaemia or feline sarcoma viruses, they make antibodies to the structural proteins of the virus. They also make antibodies to a new virus-induced cell-membrane antigen (or antigens) called FOCMA (feline oncornavirus cell membrane antigen) (Essex *et al.*, 1971*a*, *b*). Various epidemiological studies have shown that it is the ability of an animal to make FOCMA antibody successfully that is the single most important determinant of the outcome of infection with the tumour virus. Cats that respond well to this new antigen successfully resist their tumour and, in fact, are resistant to challenge by the same virus or by virus-induced tumour cells (Essex *et al.*, 1975*a*, *b*; 1976). The FOCMA antigen that is present in the cell membrane does not appear to be identical to any of the known viral structural proteins, at least in the form that we know them in the virus particle. A few years ago we found that we could transform mink cells using feline

FIG. 6.—Schematic representation of the possible results of the infection of fibroblasts by type-C viruses. (From Tooze, 1973.)

sarcoma virus, and made several non-producer feline-sarcoma-virus-transformed mink cells (Henderson, Lieber and Todaro, 1974). When these cells are tested with FOCMA antibody, they give very strong positive reactions; in contrast, cells transformed by mouse or rat sarcoma virus do not have the FOCMA antigen (Sliski *et al.*, 1977). Cells infected with just the helper leukaemia virus also lack this antigen. The antigen, then, is specific for feline-sarcoma-virus-transformed cells and not for virus infection *per se* (Sliski *et al.*, 1977). Under natural conditions the ability to produce FOCMA is the major factor in determining whether or not an animal will successfully resist its tumours. The non-producer transformed mink cells allow an opportunity to purify this specific antigen. The experiments show, further, that the antigen is coded for by the feline sarcoma virus: the antigen in the cell membrane could conceivably be a sarcoma gene product, or reflect some change in the cell membrane induced by the sarcoma gene product. Alternatively, it could represent an unusual form of one of the virus structural proteins.

When one considers what is known about the characteristics of the sarcoma gene, or the oncogene, additional properties become apparent. The first is that the gene is closely related, if not identical to, genes present in normal cellular DNA. This has been shown by studies of the sarcoma-gene-specific nucleic acid sequences and their ability to hybridize to various cellular DNAs (Stehelin *et al.*, 1976; Frankel and Fischinger, 1977). The second is that it is well conserved in evolution as demonstrated by studies using specific probes to the sarcoma-gene portion of the virus; related species have related gene sequences (Stehelin *et al.*, 1976; Frankel and Fischinger, 1977). In fact, the sarcoma-gene portion seems to be more highly conserved than the viral-gene portions of the type-C genome. Deletion mutants of chicken sarcoma viruses exist that have lost their ability to transform cells in culture. On the basis of the size of the deletion, one would estimate that the gene or genes involved in the transformation could not code for a protein with a mol. wt greater than 50,000–60,000. It appears to be produced only in very low quantities in transformed cells because, to date, attempts to isolate it have not been successful, while the other viral gene products are readily detected. Its action is to stimulate cell growth. The sarcoma viruses can add this gene from the outside and cause transformation even in heterologous species. For example, chicken sarcoma virus will transform mouse, rat and even human cells in culture, and mouse sarcoma viruses will transform cells of almost any vertebrate species. These studies would suggest that the sarcoma genes that pre-exist in normal cells may also become activated by means other than viral infection from the outside, and when they are expressed they may be able to produce an effect on the cells very similar to that produced when sarcoma genes are added by viruses from the outside.

What sorts of things fit this model? One class of substances that has to be considered is those growth hormones that stimulate cells to divide, that are present in low amounts, and that have normal physiological functions in development and perhaps throughout the entire lifetime of the animal. An example is a substance called epidermal growth factor (EGF). This is a protein with a mol. wt $\sim$ 6,000. It has 53 amino acids and has been completely sequenced (Cohen, Carpenter and Lembach, 1975). When injected into newborn mice it causes their eyelids to open earlier, or their tooth buds to erupt slightly earlier, than in the untreated animals. Using this assay, Stanley Cohen was able to purify the substance (Cohen, 1962, 1974). More recently it has been shown to be a potent stimulator of cell division in tissue culture, able to induce resting mouse or human diploid cells to begin to divide again (Armelin, 1973; Westermark, 1976). It produces its effect by binding to specific membrane receptors (Hollenberg and Cuatrecasas, 1975; Carpenter and Cohen, 1976). The growthfactor-receptor complex then somehow transmits a signal to the nucleus that leads to induction of cellular DNA synthesis. Cells that lack this specific membrane receptor are unable to be stimulated by exogenously added EGF. This substance appears to be highly conserved in evolution, as mouse EGF and human EGF cross-react strongly with one another and, in fact, compete equally well for receptor sites on the membranes of various mammalian cells (Carpenter and Cohen, 1976; Todaro, De Larco and Cohen, 1976*b*). A substance of this kind has most of the properties one would expect of a sarcoma gene product. What is the evidence that, in fact, it should be considered as a candidate oncogene product? Recent experiments from our laboratory have suggested that sarcoma-virus-transformed cells produce such a substance (see Table IX). We have, over the years, accumulated large numbers of cell lines transformed by different agents, starting with the parental mouse cell called 3T3 (Todaro and Green, 1963) or BALB/3T3 (Aaronson and Todaro, 1968). These are mouse cells that have proved useful for studies of cell transformation, because they are continuous cell lines that grow well but do not cause tumours when directly inoculated into animals, and they are very susceptible to transformation by a wide variety of agents. They have been transformed by DNA-containing viruses such as polyoma, SV40 (Todaro, Habel and Green, 1965) and herpes simplex virus (Duff and Rapp, 1975) by RNA-containing viruses such as mouse sarcoma and chicken sarcoma viruses, and by various chemical agents such as methylcholanthrene and dimethylbenzanthracene. They can also be transformed by high doses of radiation (Pollack, Aaronson and Todaro, 1970). When normal cells are compared to various transformed cells, it is seen that only mouse-sarcoma-virus-transformed cells are altered in their ability to bind exogenously added $^{125}$I-labelled epidermal growth factor ($^{125}$I-EGF) (Todaro *et al.*, 1976*b*). The mouse-sarcoma-virus (MSV)-transformed cells cannot bind exogenously added EGF, while DNA-virus-transformed cells and most of the chemically transformed cells bind labelled EGF very well. This is not the result of virus infection or virus production, because non-transforming viruses infecting and growing in these cells do not affect the level of EGF binding. It then appears to be specific for RNA-sarcoma-virus-transformed cells and not transformed cells in general.

TABLE IX.—*Evidence that Sarcoma-virus-transformed Cells Produce an EGF-related Substance*

1. Cell extracts compete for EGF-binding sites, not for gp70 or MSA-binding sites

2. Concentrated supernatants will stimulate DNA synthesis in serum-deprived 3T3 cells

3. The activity is heat-stable, acid-stable, protease-sensitive, but not precipitated with anti-EGF antibody

Several other factors have been characterized that are also polypeptides with mol. wts in the range 5–15,000; they, too, are highly conserved in evolution, interact with specific membrane receptors, and induce cells to begin to divide. Another one that we have studied is a factor called multiplication-stimulating factor (MSA). This factor is closely related to the human somatomedins. It has been isolated and purified from a rat liver-cell line that produces this substance (Dulak and Temin, 1973; Temin, Smith and Dulak, 1974; Nissley and Rechler, in press). MSA, like EGF, stimulates cell division in 3T3 cells and various normal human diploid fibroblasts and human glial cells. The sarcoma-virus-transformed cells, that are greatly altered in having lost EGF receptors, are unaltered in their levels of MSA receptors (Todaro *et al.*, 1977). Similarly, the receptors for the envelope gene product, gp70, are unchanged in transformed as opposed to normal non-producer cells (Todaro *et al.*, 1976*b*). The effect, then, seems to be specific both for the sarcoma virus and the EGF receptor system.

In our more recent studies, we have attempted to purify an EGF-related substance from sarcoma-virus-transformed cells. At the moment we have data indicating that sarcoma-virus extracts do contain a substance that will compete with the EGF receptors, but not with the gp70, MSA or NGF-specific receptors. This activity is heat-stable, acid-stable, protease-sensitive and reacts with anti-EGF antibody. Thus, we think we do have evidence that sarcoma-virus-transformed cells produce a substance much like EGF. Direct attempts to demonstrate that it is the sarcoma gene product itself are in progress.

When a variety of human tumour cells are tested for EGF receptors (Giard *et al.*, 1973) the great majority of them are found to have normal levels of receptors and, in many cases, even higher levels than human diploid fibroblasts (Fabricant, De Larco and Todaro, 1977) (see Table X). However, we have come across two human tumour lines that have no apparent EGF receptors. One is a rhabdomyosarcoma (A673) and another is a bronchogenic carcinoma (9812). The great majority of human tumour cells in culture, however, have EGF receptors. The two that appear to be lacking EGF receptors are currently being tested for the possibility that they appear to lack EGF receptors because they produce the substance or a related substance themselves. Two other fibrosarcocomas lack MSA receptors while having EGF receptors. In this case, *in vivo* transformation may be associated with perturbation of the MSA-receptor system rather than the EGF-receptor system (Todaro *et al.*, 1977). One of the human fibrosarcomas produces a factor that competes for the MSA-specific receptors and stimulates cell division of 3T3, normal rat and human diploid cells in culture.

TABLE X.—$^{125}$*I-EGF Binding to Human Fibroblasts and to Human Tumour Cells*

| | $^{125}$I-EGF bound (fmol/$10^6$ cells) |
|---|---|
| *Normal fibroblasts* | |
| Embryonic lung | 21 |
| Newborn foreskin | 29 |
| Adult skin (early passage) | 24 |
| Adult skin (late passage) | 28 |
| Adult skin (SV40 transformed) | 35 |
| *Tumour cell lines* | |
| Vulva carcinoma A431 | 203 |
| Pancreatic carcinoma A1165 | 35 |
| Renal-cell carcinoma A498 | 58 |
| Epidermoid carcinoma A388 | 67 |
| Bladder carcinoma A1663 | 38 |
| Fibrosarcoma 8387 | 52 |
| Glioblastoma A172 | 22 |
| Rhabdomyosarcoma RD | 34 |
| Rhabdomyosarcoma A673 | <0·1 |
| Bronchogenic carcinoma 9812 | <0·1 |

From the above studies, we would propose a model for cell transformation. Cell growth in a developing organism is controlled, in part, by the particular display of growth-factor receptors on the surfaces of the cells. Growth factors would be produced by cells that do not themselves respond to them. Thus, inappropriate production of growth factors, unusual responsiveness to growth factors, or some alterations in the receptors in recipient cells could serve as an endogenous stimulus for cell division. Persistent production of growth factor, or of something that interacts with the receptors in a manner like the exogenously added growth factor, would serve as a continued stimulus for cell division and inappropriate cell growth. By this model, the sarcoma virus would contain growth-factor genes, or genes that result in the production of growth factors, or a substance that interacts with the receptors so as to mimic the effect of the growth factors.

Another growth factor that may have considerable clinical interest is a substance called nerve-growth factor (NGF) (Levi-Montalcini and Angeletti, 1968). This has been shown to allow the outgrowth of dorsal-root ganglia (Levi-Montalcini, Meyer and Hamburger, 1954); specific membrane receptors have been found on nerve cells and neuroblastoma cells (Revoltella *et al.*, 1974). In some recent studies we have found that human melanoma cells have particularly high levels of receptors for NGF. Binding of

FIG. 7.—Binding of $^{125}$I-labelled nerve growth factor to melanoma cells (○) compared to its binindg to sarcoma or carcinoma cells (△).

NGF to these cells is specific for melanoma cells; there is no detectable binding to normal fibroblast or brain cells, or to sarcomas, carcinomas or lymphoid tumours (Fabricant *et al.*, 1977). So here again there appears to be a specific marker of a particular differentiated type. Fig. 7 shows the binding of $^{125}$I-labelled NGF to melanoma cells as compared to its binding to sarcoma or carcinoma cells. While the series is not at all large at the moment, it does appear that metastatic melanoma has higher levels of receptors than do primary tumours (Fabricant *et al.*, 1977). It is hard to determine whether normal melanocytes also have receptors or this represents a re-expression of embryonic antigen. Melanocytes are of neural crest origin and it may not therefore be surprising that they bind NGF as readily as they do.

Some of the potential uses of this discovery are as follows; $^{125}$I or $^{131}$I-labelled NGF may be useful as a scan to localize melanoma cells in the body, since the cell types that are known to bind labelled NGF are centrally located. "Hot spots" of NGF binding may serve as a useful early marker for primary or metastatic melanomas.

Various laboratories have reported common melanoma antigens that have appeared to cross-react between tumours from different patients (Hellström, Hellström and Warner, 1973; Hellström and Hellström, 1973; Hellström *et al.*, 1973). The possibility should be considered that the common antigen might be the NGF receptors or the NGF receptors complexed to NGF. Experiments to test whether the NGF receptors can be used as a common antigen and perhaps as a point of attack for cytotoxic tests should be considered.

If it is, in fact, the case that metastatic melanomas have higher levels of NGF receptors than the primary melanomas, and if NGF serves to stabilize the cells, thereby increasing their capacity to survive, NGF itself may play a significant natural role in determining whether or not melanomas will successfully metastasize. If this is the case, one might consider the possibility of decreasing the chances of spread with anti-NGF antibody or anti-NGF-receptor antibody, given perhaps at the time of removal of the primary tumour. Since there are model systems with mouse melanomas which do have some NGF receptors, this can be tested. Studies in the animal may give some clue as to whether this kind of approach might be feasible in man.

TABLE XI.—*Possible Functions of Genetically Transmitted Virogenes in Normal Cells*

1. Activation of oncogenic information, while inappropriate in adult tissue, plays a normal role during differentiation and development
2. The integrated virus serves to protect the species against related, more virulent infectious type-C viruses
3. Virus activation, being linked to transformation, protects the animal by altering the cell membrane. The released virus could alert the immune system making the transformed cells more susceptible to immunologic control
4. As conveyors of genetic information between species they may have had an evolutionary role. Only this group of viruses have been shown to transmit genes between germ cells of different species under natural conditions

The last table (Table XI) summarizes what I would think are the major possible explanations why these virus gene sequences have persisted so long and so well in so many vertebrate species. If they had any significant survival value to the host species, this advantage might greatly outweigh the negative effect that they would have by occasionally producing tumours either in their own host or in distant hosts. The first possibility would be that the viral gene–oncogene system has some normal role during embryonic life in differentiation and development, and perhaps this involves cell recognition or specific stimulation of certain types of cells, as well as the transmission of information from one cell to another. Whatever the reason, if this were the case, the inappropriate expression of viral or oncogenic information in adult life might be a minor factor, in an evolutionary sense, although obviously quite important to the individual involved, compared to the selective advantage, in maintaining the system.

The second kind of system in which it might be advantageous to the host would be if the endogenous, genetically transmitted virus served to protect the animal against related more virulent viruses that may be acquired from the outside or may even reside in its own genetic information. There are numerous examples in bacterial systems of integrated viruses that protect the host cells by producing immunity factors against related viruses. It may well have been, for example, that the cat ancestors that came to Africa and came in contact with the baboon virus were originally damaged by that virus. Those cats that were able successfully to integrate the virus might have been at a selective advantage relative to those that could not, because it conferred some protection against infection. Even today this is demonstrable by the fact that the baboon type-C virus will not grow in cat cells, nor will the cat virus grow in baboon cells, although their host range is quite wide. The resistance to infection appears to have persisted. It may be that one of the ways an animal has of protecting itself against a potentially harmful tumour virus is to integrate it, making it a part of its own genes and, as a consequence, acquiring a certain measure of immunity to repeated infection by the same or related viruses. This immunity could be at the level of blocking the receptors for entry into the cell, it could be intracellular at the level of preventing the DNA from integrating, or if, in fact, there are only a limited number of integration sites in the DNA, it could protect by actually occupying those sites.

The third model is an immunological one, and takes into account the finding that transformed cells, or tumour cells in general, more readily release their endogenous virus than do normal cells (Lieber, Livingston and Todaro, 1973). The activation of viral information that results in the protection of new cell-membrane antigens might actually be protective to the host, by calling attention to the cell and increasing the possibility that the immune system will reject the newly transformed cells. Following this line of reasoning, then, it might be evolutionarily advantageous for the viral gene to be linked to the transforming gene so that, when cells became transformed, if they expressed viral antigens they would be more likely to be handled by a competent immune system. In a sense, then, cancer would be "causing" viruses rather than the other way around.

The fourth, and most speculative, model is that they have served an important evolutionary role in the development of higher organisms by virtue of their ability to transmit cellular genetic information between species. That they can transmit themselves between species has been amply documented (see above). That they can pick up cellular genes has also been described (Scolnick, Maryak and Parks, 1974; Shoyab and Baluda, 1975). That this has been a major evolutionary force, however, remains only a speculation. At the point that a species becomes distinct enough from all other species that it can no longer exchange genes, its ability to change is limited to its ability to rearrange and duplicate its existing genes; it no longer has the potential to acquire genes from geographically close, but genetically distant, species. The virus provides one means of keeping species in contact with one another. The type-C viruses are admirably suited for this because they integrate with cellular DNA. When they come out again, they emerge with the possibility of having incorporated cellular genes and transmitting them to new cells, and to new species. From this perspective, the fact that they might occasionally transmit the wrong information to the wrong cell or become activated at the wrong time and in the wrong place might be a minor price for the species to have to pay in return for a system that allows them to sample information from distant parts of the body as well as from genetically distant species. The great majority of genes acquired in this fashion would be irrelevant or harmful. But if one in a billion or one in a trillion were useful to the recipient species, it might be enough to have maintained the system. The selective pressure then would be to preserve a system that allows the receipt of information from distant species. The occasional individual that receives the wrong information would not, in an evolutionary sense, be of much consequence. Viewed then, from this perspective, this group of viruses may help us to understand fundamental questions about control of cell growth and differentiation, regulation of expression and evolution. This fascinating group of viruses and cellular genes, on balance, would be helpful to the species. The occasional production of tumours by this group of viruses, or "escaped" cellular genes, would then be a pathological manifestation of a perhaps widespread, normal process. Our increased understanding of the normal functions of this system may allow us to deal better with its pathological manifestations when we encounter them.

### REFERENCES

Aaronson, S. A. & Todaro, G. J. (1968) The Basis for the Acquisition of Malignant Potential by Mouse Cells Cultivated *In vitro*. *Science, N.Y.*, **162**, 1024.

Aaronson, S. A. & Stephenson, J. R. (1976) Endogenous Type-C RNA Viruses of Mammalian Cells. *Biochim. biophys. Acta*, **458**, 323.

Armelin, H. A. (1973) Pituitary Extracts and Steroid Hormones in the Control of 3T3 Cell Growth. *Proc. natn. Acad. Sci. USA*, **70**, 2702.

Baltimore, D. (1974) Tumor Viruses. *Cold Spring Harbor Symp. Quant. Biol.*, **39**, 1187.

Benveniste, R. E. & Todaro, G. J. (1973) Homology Between Type C Viruses of Various Species as Determined by Molecular Hybridization. *Proc. natn. Acad. Sci. USA*, **70**, 3316.

Benveniste, R. E. & Todaro, G. J. (1974*a*) Multiple Divergent Copies of Endogenous Type C Virogenes in Mammalian Cells. *Nature, Lond.*, **252**, 170.

Benveniste, R. E. & Todaro, G. J. (1974*b*) Evolution of Type C Viral Genes: I. Nucleic Acid from Baboon Type C Virus as a Measure of Divergence among Primate Species. *Proc. natn. Acad. Sci. USA*, **71**, 4513.

Benveniste, R. E. & Todaro, G. J. (1975) Evolution of Type C Viral Genes. III. Preservation of Ancestral Murine Type C Viral Sequences in Pig Cellular DNA. *Proc. natn. Acad. Sci. USA*, **72**, 4090.

Benveniste, R. E. & Todaro, G. J. (1976) Evolution of Type C Viral Genes: Evidence for an Asian Origin of Man. *Nature, Lond.*, **261**, 101.

Benveniste, R. E. & Todaro, G. J. (1977) Evolution of Primate Oncornaviruses: An Endogenous Virus from Langurs (*Presbytis spp.*) with Related Virogene Sequences in Other Old World Monkeys. *Proc. natn. Acad. Sci. USA.*, **74**, 4557.

Benveniste, R. E., Sherr, C. J. & Todaro, G. J. (1975) Evolution of Type C Viral Genes: Origin of Feline Leukemia Virus. *Science, N.Y.*, **190**, 886.

Benveniste, R. E., Callahan, R., Sherr, C. J., Chapman, V. & Todaro, G. J. (1977) Two Distinct Endogenous Type C Viruses Isolated from the Asian Rodent *Mus cervicolor*: Conservation of Virogene Sequences in Related Rodent Species. *J. Virol.*, **21**, 849.

Callahan, R., Benveniste, R. E., Sherr, C. J., Schidlovsky, G. & Todaro, G. J. (1976) A New Class of Genetically Transmitted Retrovirus Isolated from *Mus cervicolor*. *Proc. natn. Acad. Sci. USA.*, **73**, 3579.

Carpenter, G. & Cohen, S. (1976) Human Epidermal Growth Factor and the Proliferation of Human Fibroblasts. *J. cell. Physiol.*, **88**, 227.

Cohen, S. (1962) Isolation of a Mouse Submaxillary Gland Protein Accelerating Incisor Eruption and Eyelid Opening in the New-born Animal. *J. biol. Chem.*, **237**, 1555.

Cohen, S. (1974) Epidermal Growth Factor: Chemical and Biological Characterization. *Recent Prog. Horm. Res.*, **30**, 533.

Cohen, S., Carpenter, G. & Lembach, K. J. (1975) Interaction of Epidermal Growth Factor (EGF) with Cultured Fibroblasts. *Adv. metab. Disord.*, **8**, 265.

Colcher, D., Heberling, R. L., Kalter, S. S. & Schlom, J. (1978) Squirrel Monkey Retrovirus: An

Endogenous Virus of a New World Primate. *J. Virol.*, **23**, 294.

DE LARCO, J. & TODARO, G. J. (1976) Membrane Receptors for Murine Leukaemia Viruses: Characterization Using the Purified Viral Envelope Glycoprotein, gp71. *Cell*, **8**, 365.

DE LARCO, J. E., RAPP, U. R. & TODARO, G. J, (1978) Cell Surface Receptors for Ecotropic MuLV: Detection and Tissue Distribution of Free Receptors *In vivo*. *Int. J. Cancer*, (In press).

DUFF, R. & RAPP, F. (1975) Quantitative Assay for Transformation of 3T3 Cells by Herpes Simplex Virus Type 2. *J. Virol.*, **15**, 490.

DULAK, N. C. & TEMIN, H. M. (1973) A Partially Purified Polypeptide Fraction from Rat Liver Cell Conditioned Medium with Multiplication-stimulating Activity for Embryo Fibroblasts. *J. cell. Physiol.*, **81**, 153.

EISENMAN, R., VOGT, V. M. & DIGGELMAN, H. (1974) Synthesis of Avian RNA Tumor Virus Structural Proteins. *Cold Spring Harbor Symp. Quant. Biol.*, **39**, 1067.

ESSEX, M., KLEIN, G., SNYDER, S. P. & HARROLD, J. B. (1971*a*) Antibody to Feline Oncorna-virus Associated Cell Membrane Antigen in Neonatal Cats. *Int. J. Cancer*, **8**, 384.

ESSEX, M., KLEIN, G., SNYDER, S. P. & HARROLD, J. B. (1971*b*) Feline Sarcoma Virus Induced Tumors: Correlation Between Humoral Antibody and Tumour Regression. *Nature, Lond.*, **233**, 195.

ESSEX, M., SLISKI, A., COTTER, S. M., JAKOWSKI, R. M. & HARDY, W. D., JR (1975*a*) Immunosurveillance of Naturally Occurring Feline Leukemia. *Science, N.Y.*, **190**, 790.

ESSEX, M., COTTER, S. M., CARPENTER, J. L., HARDY, W. D., JR, HESS, P. W., JARRETT, W., SCHALLER, J. & YOHN, D. S. (1975*b*) Feline Oncornavirus-associated Cell Membrane Antigen. II. Antibody Titers in Healthy Cats from Household and Laboratory Colony Environments. *J. natn. Cancer Inst.*, **54**, 631.

ESSEX, M., SLISKI, A., HARDY, W. D., JR & COTTER, S. M. (1976) Immune Response to Leukaemia Virus and Tumor-associated Antigens in Cats. *Cancer Res.*, **36**, 640.

FABRICANT, R. N., DE LARCO, J. E. & TODARO, G. J. (1977) Nerve Growth Factor Receptors on Human Melanoma Cells in Culture. *Proc. natn. Acad. Sci. USA*, **74**, 565.

FISCHINGER, P. J., PEEBLES, P. T., NOMURA, S. & HAAPALA, D. K. (1973) Isolation of an RD-114-like Oncornavirus from a Cat Cell Line. *J. Virol.* **11**, 978.

FRANKEL, A. F. & FISCHINGER, P. J. (1977) Rate of Divergence of Cellular Sequences Homologous to Segments of Moloney Sarcoma Virus. *J. Virol.*, **21**, 153.

GIARD, D. J., AARONSON, S. A., TODARO, G. J., ARNSTEIN, P., KERSEY, J. H., DOSIK, H. & PARKS, W. P. (1973) *In vitro* Cultivation of Human Tumors: Establishment of Cell Lines Derived from a Series of Solid Tumors. *J. natn. Cancer. Inst.*, **51**, 1417.

HARDY, W. D., JR, OLD, L. J., HESS, P. W., ESSEX, M. & COTTER, S. (1973) Horizontal Transmission of Feline Leukaemia Virus. *Nature, Lond.*, **244**, 266.

HEBERLING, R. L., BARKER, S. T., KALTER, S. S., SMITH, G. C. & HELMKE, R. J. (1977) Oncornavirus: Isolation from a Squirrel Monkey (*Saimiri sciureus*) Lung Culture. *Science, N.Y.*, **195**, 289.

HELLSTRÖM, I. G., HELLSTRÖM, K. E. & WARNER, G. A. (1973) Increase of Lymphocyte-mediated Tumor-cell Destruction by Certain Patient Sera. *Int. J. Cancer*, **12**, 348.

HELLSTRÖM, I. G. & HELLSTRÖM, K. E. (1973) Some Recent Studies on Cellular Immunity to Human Melanomas. *Fed. Proc.*, **32**, 156.

HELLSTRÖM, I. G., WARNER, A., HELLSTRÖM, K. E. & SJÖGREN, H. O. (1973) Sequential Studies on Cell-mediated Tumor Immunity and Blocking Serum Activity in Ten Patients with Malignant Melanoma. *Int. J. Cancer*, **11**, 280.

HENDERSON, I. C., LIEBER, M. M. & TODARO, G. J. (1974) Mink Cell Line Mv1Lu (CCL 64): Focus Formation and the Generation of "Nonproducer" Transformed Cell Lines with Murine and Feline Sarcoma Viruses. *Virology*, **60**, 282.

HOLLENBERG, M. D. & CUATRECASAS, P. (1975) Insulin and Epidermal Growth Factor: Human Fibroblast Receptors Related to Deoxyribonucleic Acid Synthesis and Amino Acid Uptake. *J. biol. Chem.*, **250**, 3845.

HUEBNER, R. J. & TODARO, G. J. (1969) Oncogenes of RNA Tumor Viruses as Determinants of Cancer. *Proc. natn. Acad. Sci. USA*, **64**, 1087.

KAWAKAMI, T. G. & BUCKLEY, P. M. (1974) Antigenic Studies in Gibbon Type-C Viruses. *Transplantation Proc.*, **6**, 193.

KAWAKAMI, T. G., HUFF, S. D., BUCKLEY, P. M., DUNGWORTH, D. L., SNYDER, S. P. & GILDEN, R. V. (1972) C-type Virus Associated with Gibbon Lymphosarcoma. *Nature, New Biol.*, **235**, 170.

LERNER, R. A., WILSON, C. B., DEL VILLANO, B. C., MCCONAHEY, P. J. & DIXON, F. J. (1976) Endogenous Oncornaviral Gene Expression in Adult and Fetal Mice: Quantitative, Histologic, and Physiologic Studies of the Major Viral Glycoprotein gp70. *J. exp. Med.*, **143**, 151.

LEVI-MONTALCINI, R. & ANGELETTI, P. U. (1968) Nerve Growth Factor. *Physiol. Rev.*, **48**, 534.

LEVI-MONTALCINI, R., MEYER, A. & HAMBURGER, V. (1954). *In vivo* Experiments on the Effects of Mouse Sarcoma 180 and 37 on the Spinal Cord and Sympathetic Ganglia of the Chick Embryo. *Cancer Res.*, **14**, 49.

LIEBER, M. M., LIVINGSTON, D. M. & TODARO, G. J. (1973) Superinduction of Endogenous Type C Virus by 5-bromodeoxyuridine from Transformed Mouse Cells. *Science, N.Y.*, **181**, 443.

LIEBER, M. M., SHERR, C. J., TODARO, G. J., BENVENISTE, R. E., CALLAHAN, R. & COON, H. G. (1975) Isolation from the Asian Mouse *Mus caroli* of an Endogenous Type C Virus Related to Infectious Primate Type C Viruses. *Proc. natn. Acad. Sci. USA*, **72**, 2315.

LIVINGSTON, D. M. & TODARO, G. J. (1973) Endogenous Type C Virus from a Cat Cell Clone with Properties Distinct from Previously Described Feline Type C Virus. *Virology*, **53**, 142.

MCALLISTER, R. M., NICOLSON, M., GARDNER, M. B., RONGEY, R. W., RASHEED, S., SARMA, P. S., HUEBNER, R. J., HATANAKA, M., OROSZLAN. S., GILDEN, R. V., KABIGTING, A. & VERNON, L. (1972) C-type Virus Released from Cultured Human Rhabdomyosarcoma Cells. *Nature, Lond.*, **235**, 3.

NISSLEY, S. P. & RECHLER, M. M. (1976). NCI Monograph: The Third Decennial Tissue Culture Association Research Conference, Lake Placid, New York, Sept. 13–17 (In press).

Olson, C., Miller, L. D., Miller, J. M. & Hoss, H. E. (1972) Transmission of Lymphosarcoma from Cattle to Sheep. *J. natn. Cancer Inst.*, **49**, 1463.

Pollack, E. J., Aaronson, S. A. & Todaro, G. J. (1970) X-irradiation of BALB/3T3: Sarcoma-forming Ability and Virus Induction. *Int. J. Radiat. Biol.*, **17**, 97.

Revoltella, R., Bertolini, L., Pediconi, M. & Vignetti, R. (1974) Specific Binding of Nerve Growth Factor (NGF) by Murine C 1300 Neuroblastoma Cells. *J. exp. Med.*, **140**, 437.

Rice, N. R. & Straus, N. A. (1973) Relatedness of Mouse Satellite Deoxyribonucleic Acid to Deoxyribonucleic Acid of Various *Mus* Species. *Proc. natn. Acad. Sci. USA*, **70**, 3546.

Scolnick, E. M., Maryak, J. M. & Parks, W. P. (1974) Levels of Rat Cellular RNA Homologous to Either Kirsten Sarcoma Virus or Rat Type-C Virus in Cell Lines Derived from Osborne-Mendel Rats. *J. Virol.*, **14**, 1435.

Sen, A. & Todaro, G. J. (1977) The Genome-associated, Specific RNA Binding Proteins of Avian and Mammalian Type C Viruses. *Cell*, **10**, 91.

Sen, A., Sherr, C. J. & Todaro, G. J. (1976) Specific Binding of the Type C Viral Core Protein p12 with Purified Viral RNA. *Cell*, **7**, 21.

Sherr, C. J., Benveniste, R. E. & Todaro, G. J. (1974) Type C Viral Expression in Primate Tissues. *Proc. natn. Acad. Sci. USA*, **71**, 3721.

Sherr, C. J., Benveniste, R. E., Lieber, M. M. & Todaro, G. J. (1976) Type C Viruses from Kirsten Sarcoma-transformed Mink Cells Co-cultivated with Primate Cells and Expressing p30 Antigens Related to Feline Leukaemia Virus. *J. Virol.*, **19**, 346.

Sherr, C. J., Benveniste, R. E. & Todaro, G J. (1978) Endogenous Mink (*Mustela vison*) Type C Virus Isolated from Sarcoma Virus-transformed Mink Cells. *J. Virol.* (In press).

Shoyab, M. & Baluda, M. A. (1975) Homology Between Avian Oncornavirus RNAs and DNA from Several Avian Species. *J. Virol.*, **16**, 1492.

Sliski, A. H., Essex, M., Meyer, C. & Todaro, G. J. (1977) Feline Oncornavirus Associated Cell Membrane Antigen (FOCMA): Expression in Feline Sarcoma Virus Transformed Non-producer Mink Cells. *Science, N.Y.*, **196**, 1336.

Stehelin, D., Guntaka, R. V., Varmus, H. E. & Bishop, J. M. (1976) Purification of DNA Complementary to Nucleotide Sequences Required for Neoplastic Transformation of Fibroblasts by Avian Sarcoma Viruses. *J. molec. Biol.*, **101**, 349.

Temin, H. M., Smith, G. L. & Dulak, N. C. (1974) In *Control of Proliferation in Animal Cells*. Ed. B. Clarkson and R. Baserga. New York: Cold Spring Harbor Laboratory, p. 19.

Theilen, G. H., Gould, D., Fowler, M. & Dungworth, D. L. (1971) C-type Virus in Tumor Tissue of a Woolly Monkey (*Lagothrix ssp.*) with Fibrosarcoma. *J. natn. Cancer Inst.*, **47**, 881.

Todaro, G. J. & Green, H. (1963) Quantitative Studies of the Growth of Mouse Embryo Cells in Culture and Their Development into Established Lines. *J. cell. Biol.*, **17**, 299.

Todaro, G. J., Habel, K. & Green, H. (1965) Antigenic and Cultural Properties of Cells Doubly Transformed by Polyoma and SV40. *Virology*, **27**, 179.

Todaro, G. J., Tevethia, S. S. & Melnick, J. L. (1973) Isolation of an RD-114-related Type-C Virus from Feline Sarcoma Virus-transformed Baboon Cells. *Intervirology*, **1**, 399.

Todaro, G. J., Sherr, C. J., Benveniste, R. E., Lieber, M. M. & Melnick, J. L. (1974) Type C Viruses of Baboons: Isolation from Normal Cell Cultures. *Cell*. **2**, 55.

Todaro, G. J., Lieber, M. M., Benveniste, R. E., Sherr. C. J., Gibbs, C. J., Jr & Gajdusek, D. C. (1975) Infectious Primate Type C Viruses; Three Isolates Belonging to a New Subgroup from the Brains of Normal Gibbons. *Virology*, **67**, 335.

Todaro, G. J., Sherr, C. J. & Benveniste, R. E. (1976*a*) Baboons and Their Close Relatives are Unusual Among Primates in Their Ability to Release Nondefective Endogenous Type C Viruses. *Virology*, **72**, 278.

Todaro, G. J., De Larco, J. E. & Cohen, S. (1976*b*) Transformation by Murine and Feline Sarcoma Viruses Specifically Blocks Binding of Epidermal Growth Factor to Cells. *Nature, Lond.*, **264**, 26.

Todaro, G. J., Benveniste, R. E., Callahan, R. E., Lieber, M. M. & Sherr, C. J. (1974) Endogenous Primate and Feline Type C Viruses. *Cold Spring Harbor Symp. Quant. Biol.*, **39**, 1159.

Todaro, G. J., De Larco, J. E., Nissley, S. P. & Rechler, M. M. (1977) MSA and EGF Receptors on Sarcoma Virus Transformed Cells and Human Fibrosarcoma Cells in Culture. *Nature, Lond.*, **267**, 526.

Todaro, G. J., Benveniste, R. E., Sherr, C. J., Schlom, J., Schidlovsky, G. & Stephenson, J. R. (1977) Isolation and Characterization of a New Type D Retrovirus from the Asian Primate, *Presbytis obscurus* (Spectacled Langur). *Virology*, **84**, 189.

Tooze, J., Ed. (1973) *The Molecular Biology of Tumour Viruses*. New York: Cold Spring Harbor Laboratory, p. 619.

Vogt, V. M., Eisenman, R. & Diggelman, H. (1975) Generation of Avian Myeloblastosis Virus Structural Protein by Proteolytic Cleavage of a Precursor Polypeptide. *J. mol. Biol.*, **96**, 471.

Weiss, R. A. (1976) Receptors for RNA Tumor Viruses. In *Cell Membrane Receptors for Viruses, Antigens and Antibodies, Polypeptide Hormones, and Small Molecules*. Ed. R. F. Beers, Jr and E. G. Bassett. New York: Raven Press, p. 237.

Westermark, B. (1976) Density Dependent Proliferation of Human Glial Cells Stimulated by Epidermal Growth Factor. *Biochem. biophys. Res. Comm.*, **69**, 304.

Wolfe, L. G., Deinhardt, F., Theilen, G. H., Rabin, H., Kawakami, T. G. & Bustad, L. K. (1971) Induction of Tumors in Marmoset Monkeys by Simian Sarcoma Virus, Type I (*Lagothrix*): A Preliminary Report. *J. natn. Cancer Inst.*, **47**, 1115.